# Virus-encoded metabolism may support environmental stress adaptation of microbial hosts in an estuarine hypoxic zone

**DOI:** 10.3389/fmicb.2026.1785655

**Published:** 2026-03-24

**Authors:** Mengqi Sun, Zelin Lei, Bingbing Li, Shu-Hong Gao, Lu Fan

**Affiliations:** 1 Department of Ocean Science and Engineering, Southern University of Science and Technology (SUSTech), Shenzhen, Guangdong, China; 2 State Key Laboratory of Urban Water Resource and Environment, School of Civil & Environmental Engineering, Harbin Institute of Technology Shenzhen, Shenzhen, Guangdong, China

**Keywords:** auxiliary metabolic gene, estuarine hypoxic zone, metagenomics, microbial community, viral community, virus-host relationship

## Abstract

Hypoxic zones in estuaries threaten the ecological balance and the productivity in coastal areas. However, it is poorly understood how viruses regulate metabolic processes of their microbial hosts to adapt to the hypoxic environment, and consequently impact the biogeochemical cycles in hypoxic zones. In this study, the diversity and functional potentials of the bacterial, archaeal and viral communities of a hypoxic zone at the Pearl River Estuary was characterized along with local environmental factors, with a particular focus on viral auxiliary metabolic genes (AMGs). The viral community derived from the virion fraction and the cellular fraction of the seawater were distinctly different, with the cellular fraction generating fewer unique viruses, but more types of AMGs. Overall, more AMGs were identified in samples with higher dissolved oxygen levels. Globally conserved AMGs were infrequently observed in the current samples, suggesting a certain level of adaptation of AMGs to the local environment. There were strong correlations in abundances among cyanobacteria, cyanophages, and photosynthesis AMGs, suggesting potential viral participation in estuarine primary production. Many AMGs involved in nutrient limitation endurance were found, potentially assisting their host with phosphorus, iron and B family vitamin shortages. Although putative hosts were predicted for the viruses, the functionality of their AMGs appears to be a better predictor of their distribution than the hosts they infect. Our study provides a functional insight into the viral community in poorly researched estuarine hypoxic zones, and sheds light on the potential interactions of viruses with their microbial hosts for co-adaptation to this unique environment.

## Introduction

Coastal and estuarine hypoxia is often caused by anthropogenically induced eutrophication, creating so-called “oxygen-limited dead zones.” Globally, coastal hypoxic zones are estimated to be expanding at a rate of 5.5% per year (Rabalais et al., 2014; Fennel and Testa, 2019).

Waters are generally considered to be hypoxic if dissolved oxygen (DO) levels are below 2 mg/L (Li et al., 2018). In estuarine hypoxic zones, if organic matter of blooming algae is not consumed by planktonic microbes in a timely matter, it will sink down to the sediment and degrade there by exhausting oxygen, enhancing bottom water hypoxia (Winget et al., 2011; Crump and Bowen, 2024). When dissolved oxygen levels are sufficiently low, most multicellular consumers cannot survive and microbial activity dominates estuarine energy metabolism instead of flowing organic matter to higher trophic levels (Damashek and Francis, 2018; Diaz and Rosenberg, 2008). Unique microbial communities are often formed in estuarine hypoxic zones, and the complex environmental factors in estuaries often give rise to abundant and diverse viral populations (Sun et al., 2021; Zhang et al., 2021). However, most studies on viral activity in estuarine hypoxic zones are focused on overall viral abundance and production (Winget et al., 2011; Köstner et al., 2017; Choi et al., 2003); the composition and function of hypoxic estuarine viral communities is still largely unknown.

Auxiliary metabolic genes (AMGs) are viral-encoded genes that, when expressed in host cells, either redirect host metabolism to maximize viral reproduction, or augment host metabolism to increase the survival rates of both the viruses and their hosts (Warwick-Dugdale et al., 2019). It has been estimated that as much as 19–38% of marine viruses contain AMGs, and those AMGs tend to target key steps in a metabolic pathway of the hosts, implying that their expression may play a significant role in marine nutrient cycling (Tian et al., 2024). In the marine environment, many AMGs potentially participating in carbon, nitrogen and sulfur metabolism have been found, suggesting the role of viral AMGs in elemental cycling (Roux et al., 2016; Long et al., 2021; Luo et al., 2022). In oxygen minimum zones (OMZs) of open oceans, which are also depleted of oxygen but are formed by different mechanisms compared to estuarine hypoxic zones, viruses encoding AMGs may modulate microbial N cycling processes and may contribute to overall nitrogen loss in the ocean (Gazitúa et al., 2021), or assist their hosts in surviving environmental stress caused by hypoxia (Jurgensen et al., 2022). The distinct functional profiles of AMGs in different environments may be due to the selection for genes responding to metabolic bottlenecks in that particular environment (Breitbart et al., 2018). Compared to marine OMZs, little is known about the viral community and its AMG profile in estuarine hypoxic zones. This study provides insight into virus-host interactions in an estuarine hypoxic zone and the potential functional role of viruses in an environmental context.

Pearl River Estuary (PRE), a subtropical estuary surrounded by several megacities, is heavily impacted by human activity, with its bottom waters susceptible to summer hypoxia that is relatively well-documented (Zhang et al., 2022; Li et al., 2020). In this study, we document the diversity and function of the microbial community in and around the estuarine hypoxic zone at PRE, and particularly explore how viral AMGs can potentially assist their hosts amidst environmental stress in this deoxygenated environment.

## Materials and methods

### Sample collection

**Figure 1:**
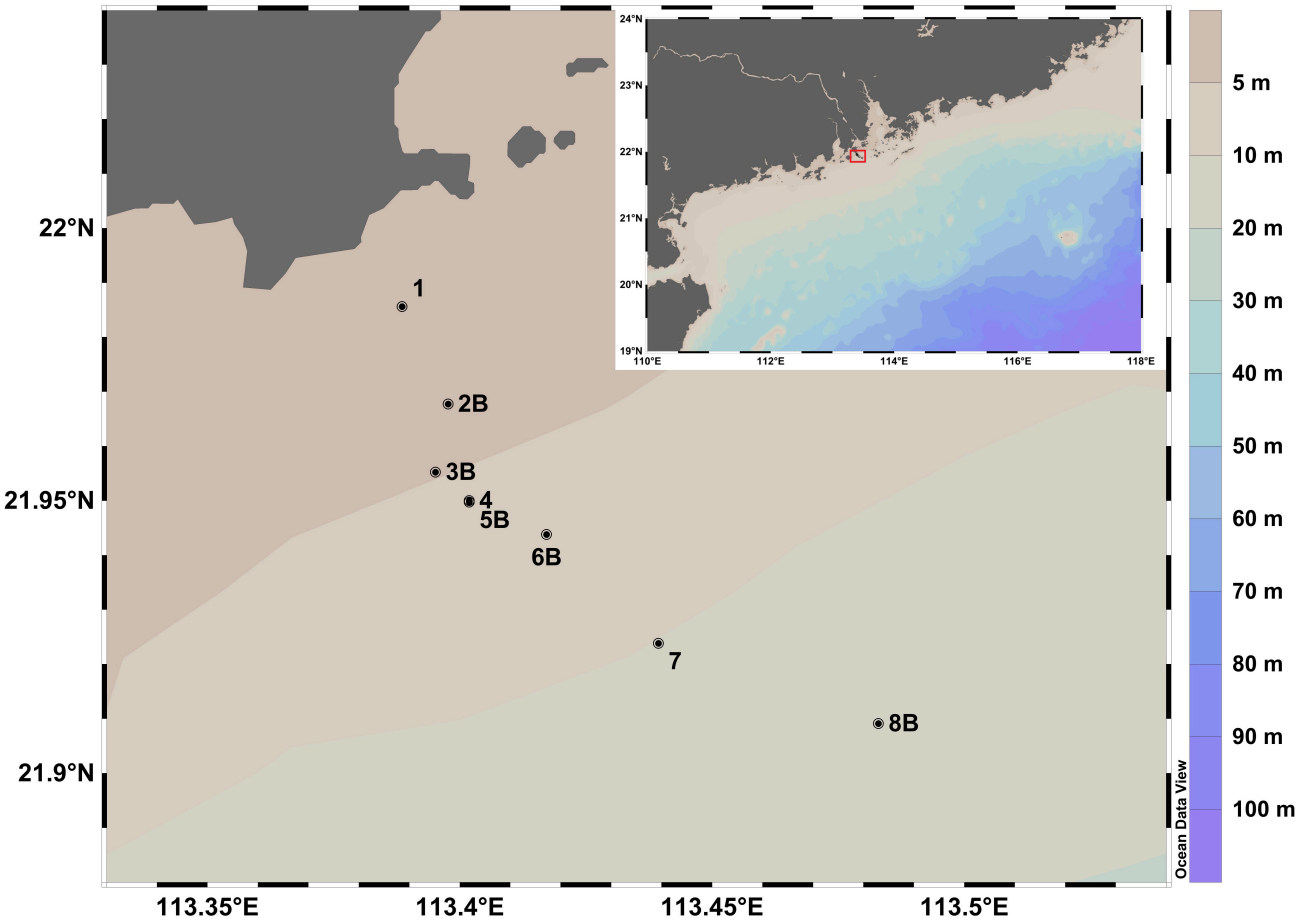
Sample map at the Pearl River estuary hypoxic zone at Jinwan Bay, on September 02, 2022. Samples 4 and 5 were taken at the same site at different depths. The “B” suffix of sample names indicates that this is a bottom water sample.

Seawater samples were collected at the hypoxic zone off the western coast of the PRE on September 2, 2022. A total of eight samples were taken at different depths covering the water columns in, above, beside and distant from the hypoxic zone (Figure 1). Sample locations, depths and other metadata are visualized using Ocean Data View (Schlitzer, 2025). See Supplementary Information for an explanation of sampling representativity.

### Measurements of environmental conditions

Dissolved oxygen was measured using a portable dissolved oxygen meter (Shanghai Measuretech Instrument Co., Ltd, JPB-607A). Salinity was measured using a refractometer (Deli, DL339103). Aquatic chemical concentrations including nitrate, nitrite, ammonia, inorganic phosphorus, sulfate and sulfide were measured using an automated discrete analyzer (DeChem-Tech, Cleverchem), while total phosphorus levels were measured using the neutral digestion method with sodium persulfate (Ma et al., 2017). Three field replicates were taken for the dissolved oxygen and salinity measurements, while three technical replicates were taken for the chemical concentrations.

### Biological sample preparation

Seawater with volumes of 5–10 L were collected at each site with 5 L vertical water samplers. Water samples were first passed through a 3-μm membrane filter (Merck Millipore), then a 0.2 μm membrane filter (Merck Millipore), with the resulting filters stored at −80 °C. FeCl_3_ (Macklin) flocculation was used to concentrate virus particles from the 0.2 μm filtrate, which were collected on a 0.8-μm filter and stored at 4 °C (John et al., 2011). Due to the constraints of sampling conditions available, biological replicates were unable to be taken.

### Virion counting

Samples from 0.2 μm filtrate were treated with DNase I (Sangon Biotech Co., Ltd.) at 37 °C for 1 h using the Sullivan Lab protocol (Hurwitz et al., 2013a). The resulting samples were filtered through 0.02-μm Anodisc filters (Whatman) and stained with SYBR Gold (Sangon Biotech Co., Ltd.; Chen et al., 2001). Virion particles were counted manually under an epifluorecence microscope (Nikon Corporation Tokyo, Eclipse Ni-U).

### DNA extraction and sequencing

DNA of the microbial cellular fraction (particulate size 0.2–3 μm) was extracted from the 0.2 μm filter using the FastDNA^®^ SPIN kit for soil (MP Biomedicals, Solon, USA). DNA was hybrid sequenced using the NovaSeq 6000 platform (Illumina) and the MinION platform (Oxford Nanopore), respectively. Nanopore sequences were prepared using the SQK-LSK109 ligation sequencing kit and sequenced with the R9.4 protocol.

Virion particles were resuspended from the 0.8-μm filters using ascorbate-EDTA buffer (0.25-M ascorbic acid, 0.2-M Mg2EDTA, and pH 6–7) and concentrated using 100 KDa Amicon filters. Samples were digested with DNase I (Sangon Biotech Co., Ltd.) at 37 °C for 1 h at 1 U/ul working dilution and Proteinase K (Sangon Biotech Co., Ltd.). DNA was then extracted using the phenol-chloroform/isoamyl (Macklin) method (Sambrook and Russell, 2006). Viral DNA was amplified using the Illustra™ Ready-To-Go™ GenomiPhi™ V3 DNA Amplification Kit, then sequenced using the Illumina NovaSeq 6000 platform at Magigen Biotechnology Co., Ltd (Guangzhou, China).

### Metagenome-assembled genome (MAG) generation and analysis

Sequence processing methods are summarized in Supplementary Figure S1. Illumina reads were trimmed using the Read_qc module of MetaWRAP (v1.3; Uritskiy et al., 2018). Illumina and Nanopore reads were hybrid assembled using Unicycler (v0.5.0; Wick et al., 2017). Clean Illumina reads were mapped to the initial assembly using bwa (v0.7.18), and the resulting BAM files were used to polish the assembly using Pilon (v1.24; Li and Durbin, 2009; Walker et al., 2014). The consolidated contigs were binned and refined using the binning module of MetaWRAP, with CheckM parameters “-c 70 -x 5.”

Taxonomy of the bins were determined using GTDB-TK (v2.3.2; Chaumeil et al., 2020). Phylogenetic trees of the bins were generated with IQTREE (v2.2.5), using the alignments from GTDB-TK (Nguyen et al., 2015). Functional annotations of bins were performed using DRAM (v1.5.0). For abundance analysis, low complexity regions of the bins were identified using Metaxa2 (v2.2), Barrnap (v0.9; https://github.com/tseemann/barrnap), tRNAscan-SE (2.0), and DustMasker (NCBI toolkit 22.0.0), and masked using Bedtools (2.31.1; Bengtsson-Palme et al., 2015; Chan et al., 2021; Quinlan and Hall, 2010). Illumina reads were mapped to bins using bowtie2 (2.5.1; Langmead and Salzberg, 2012). Total RPKM of each bin was calculated using this formula: total RPKM of each bin = (total reads in each bin) × 10^9^/(total length of each bin × total reads in sample).

The archaea bin tree and the top 50 most abundant bacteria bin tree were visualized in iTOL (7.0; Letunic and Bork, 2019). Heatmaps of the log10 (RPKM) abundance of the bins were visualized using pheatmap in R.

### Viral population generation and analysis

Reads from the < 0.2 μm fraction were trimmed using the Read_qc module of MetaWRAP, and assembled using metaSPAdes (SPAdes v3.15.5) using the default parameters (Uritskiy et al., 2018; Nurk et al., 2017). Viral sequences were identified from the < 0.2 μm fraction assembly and the 0.2–3 μm fraction polished assembly, respectively, using the following approach. Contigs over the length of 5 Kb were retained, and viral contigs were identified using VirSorter2 (2.2.4; score > 0.5) and DeepVirFinder (1.0; score > 0.7 and *p* < 0.05; Guo et al., 2021; Ren et al., 2020). Contigs were considered to be viral if they meet any of these criteria: (1) Score > 0.9 by VirSorter2; OR (2) Score > 0.9 and *p* < 0.05 by DeepVirFinder; OR (3) Score > 0.5 by VirSorter2 and also score > 0.7 and *p* < 0.05 by DeepVirFinder. Viral contigs were clustered using the “Rapid genome clustering based on pairwise ANI” protocol (95% average nucleotide identity and 85% alignment fraction) provided in the “Supporting code” section in CheckV (v1.0.3), and the longest contig of each cluster was extracted to produce a non-redundant set of viral populations (equivalent to virus OTUs; Nayfach et al., 2021). Viral populations were annotated and AMGs were predicted using DRAMv (DRAM v1.5.0) using the default parameters (Shaffer et al., 2020). DRAMv uses a score system to evaluate whether the putative AMG is likely to be a false positive resulting from host contamination, and only categories 1–3 (AMGs flanked by two or more viral like genes) were taken into consideration. Viruses identified from the viral and microbial cellular fraction were combined to form a dataset of total non-redundant viral populations.

Viral populations were clustered with reference genomes using vConTACT2 (0.11.3; –rel-mode “Diamond” –db “ProkaryoticViralRefSeq201-Merged” –pcs-mode MCL –vcs-mode ClusterONE) and the resulting cluster network was visualized using Cytoscape (Bolduc et al., 2017; Shannon et al., 2003).

### Viral taxonomy and abundance analysis

For viral abundance analysis, clean Illumina DNA reads from the viral and microbial cellular fractions were mapped to viral populations using bbmap at 90% identity, results were normalized to KPKG (total mapped nucleotides (KB) per KB of genome per GB of metagenome) (Bushnell, 2014; Martinez-Hernandez et al., 2017). KPKG is used as a proxy for relative abundance.

To identify the top 30 most abundant viral populations, predicted proteins were aligned with Viral Refseq (release 224) using BLASTP (identity> 30%, bitscore > 50, e-value < 0.001), then for each viral population, the match with the lowest e-value was determined to be the best hit (Altschul et al., 1990). Accession numbers in the BLASTP results were connected to NCBI taxonomy using taxonomizr in R (Sherrill-Mix, 2023); if the NCBI taxonomy species name and genome name have a conflict, the genome name was selected. Relative abundance of the top 30 viral populations was visualized using ggplot2 (Ginestet, 2011).

### Host prediction for viral sequences

Hosts of the viruses were predicted using iPHoP (v1.3.3; Roux et al., 2023). First, the hosts of the viral populations were initially predicted using the standard iPHoP database. To avoid self-hits and achieve accurate virus-host predictions, the host database must be free of viral sequences. Contigs containing lingering viral sequences were removed from the microbial bins (MAGs) using the same viral identification pipeline described above. The subsequent virus-free MAGs were run through the *de novo* pipeline of GTDB-tk, and the taxonomic results were added to the iPHoP database, which was used to predict the hosts of the viral populations again (confidence score > 90). The top match of each virus was determined to be its predicted host. If the predicted host is a microbial bin from the microbial samples in this study instead of an iPHoP database hit, it is considered a “local” virus-host pair. Local virus-host pairs were visualized using Cytoscape.

To search for connections between the hosts viruses infect and the AMGs they carry, viruses with both a host prediction and containing AMGs were counted and visualized using a dotplot in ggplot2.

### AMG identification and curation

Putative AMGs were predicted with DRAMv from the viral populations from both the virion and microbial cellular factions. Then the AMGs were manually curated according to previously proposed standards (Luo et al., 2022; Pratama et al., 2021; Martin et al., 2025). Namely, genes assigned to nucleotide metabolism, modification of viral components, viral invasion, ribosomal proteins, or transcriptional/translational regulators were removed, as they were assumed to primarily serve the virus's own assembly/invasion needs instead of being of auxiliary metabolic interest. A list of keywords for removal of genes from AMGs can be found in Supplementary Table S1. Although some studies consider nucleotide metabolism genes to be AMGs (Tian et al., 2024), they are not included in this study, as we are primarily interested in the variation of AMGs in the context of environmental selection, as opposed to the virus's own genomic assembly needs (Martin et al., 2025). The resulting collection of AMGs were then manually sorted into 13 metabolic categories. The KPKG values of the contig containing the AMG were used as a proxy for AMG relative abundance.

To explain the effect of environmental and chemical factors (Supplementary Table S2) on relative abundance of each AMG, redundancy analysis (RDA) was performed using the vegan package in R and visualized with ggplot2 (Ginestet, 2011; Oksanen et al., 2025). Since the number of environmental factors is limited, to avoid overfitting and multicolinearity among response variables, only AMGs related to nutrient, photosynthesis and stress response are used in the analysis.

## Results and discussion

### Sample processing

A transect across the hypoxic zone at the mouth of the PRE was captured with eight samples, with sampling depth ranging from 5 to 15 m, and dissolved oxygen values ranging from 1.1 to 8.5 mg/L (Figure 1, Supplementary Table S2). Sulfide concentrations were below detection levels, while total phosphorus levels were relatively higher in hypoxic samples 5B and 6B compared to other samples (Supplementary Table S2). Viral counts of most samples ranged between 10^7^ and 10^8^ VLP/ml, as is expected in marine environments (Wigington et al., 2016; Supplementary Figure S2).

Due to the small size and low levels of genetic material in virion particles, only five viral samples yielded enough DNA for Illumina sequencing (Table 1). An average of 11.1 GB of raw sequence data were produced from each virion fraction sample (< 0.2 μm). Microbial cellular fraction samples (0.2–3 μm) produced 11.6 GB of raw Illumina sequence data and 13.7 GB of raw nanopore sequence data (Supplementary Table S3). An average of 72.4% of contigs larger than 5 kb in each virion fraction sample were identified as viral, while an average of 17.4% of contigs in each microbial cellular fraction sample were identified as viral (Table 1).

**Table 1:** Virus identification results and AMG counts.

**Sample**	**Raw Illumina reads (Mb)**	**# Contigs > 5 kb**	**# Viral contigs**	**# Non-redundant viral populations**	**# Combined non-redundant viral populations**
M1	75.69	5,868	1,531	Microbial samples: 8,472	19,370
M2B	75.73	10,147	2,367		
M3B	79.1	13,042	2,410		
M4	79.78	15,573	2,949		
M5B	90.68	13,692	2,278		
M6B	71.15	14,211	1,178		
M7	71.42	8,340	877		
M8B	74.29	11,093	1,904		
V1	72.32	2,675	2,287	Viral samples: 11,907	
V3B	72.05	4,608	3,794		
V6B	67.46	384	140		
V7	85.29	7,218	5,973		
V8B	74.14	1,639	1,228		

Samples from the 0.2–3 μm fraction start with “M” for microbial, samples from the < 0.2 μm fraction start with “V” for viral; bottom water samples include “B.” Viral populations are clustered viral contigs with redundancy removed, equivalent to virus OTUs.

### Microbes are more abundant in higher oxygen samples

A total of 91 metagenome-assembled genomes (MAGs) were identified from the microbial cellular fraction, including 83 bacterial MAGs and eight archaea MAGs (completeness ≥70%, contamination ≤ 5%; Supplementary Figure S3). Overall, samples of higher dissolved oxygen levels have relatively higher microbial abundance, with samples 1 and 8B (spanning the width of the hypoxic zone) showing the most abundant microbial populations (Supplementary Figure S4). Most abundant MAGs were more abundant in samples 1 and 8B with relatively higher dissolved oxygen levels, except for a *Verrucomicrobiae*, an HTCC2207 bacterium, a *Nitrosopumilus*, and a *Rhodobacterceae* that were more abundant further away from the coast, presumably due to their marine origins (Supplementary Figure S4).

### Viral populations differ across cellular and virion fractions

**Figure 2:**
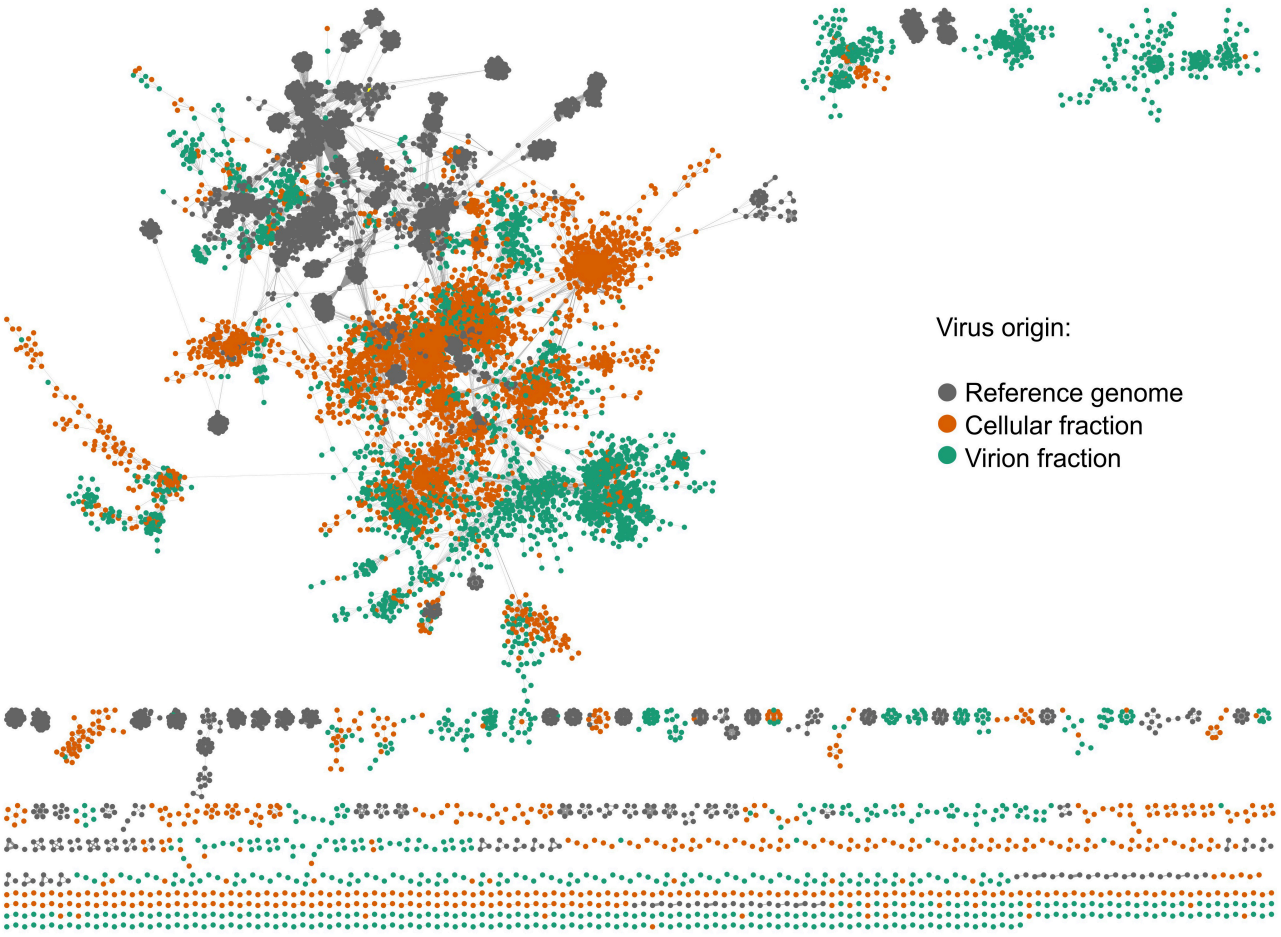
Hypoxic zone virus cluster network. Gray dots are reference virus genomes from vContact, orange dots are viruses originating from the 0.2–3 μm fraction, green dots are viruses originating from the < 0.2 μm fraction.

A total of 19,370 viral populations were identified from the virion and microbial cellular fractions after removing redundancy (Table 1). Cluster network analysis of the viral populations with reference genome shows most local viral populations forming distinct clusters away from the RefSeq genomes (Figure 2). While the populations arising from the viral and microbial cellular fractions were more closely connected to each other compared to the reference genomes, they also formed distinct clusters from each other, with populations derived from the virion fraction showing slightly more diversity (Figure 2). To examine the impact of oxygen levels on viral community structure, linear correlation of dissolved oxygen levels and the abundance of the top 5,000 viral populations was conducted, and no statistically significant correlation was found.

To visualize the similarity of samples based on viral or microbial abundance, a non-metric multidimensional scaling (NMDS) based on Bray-Curtis dissimilarity matrices was plotted (Ginestet, 2011; Oksanen et al., 2025). Curiously, NMDS analysis shows that the microbes in the microbial cellular fraction and viral populations in the microbial cellular fraction follow the same distribution in terms of sample similarity (Supplementary Figures S5A, S5B). Meanwhile, viral samples follow a different pattern, with sample V6B showing the greatest dissimilarity from the other samples (Supplementary Figure S5C). Since sample 6B has the lowest dissolved oxygen, this suggests that hypoxic conditions may have a larger impact on dispersed viral communities compared to microbial communities or host-associated viral communities. Combined NMDS visualization of viral abundance in the microbial cellular fraction and viral fraction resulted in significantly higher dissimilarity in viral fraction compared to the microbial cellular fraction (Supplementary Figure S5D). This is most like a result of different sample processing methods, but may also be reflective of the higher genetic diversity of dispersed viruses vs. host associated viruses.

**Figure 3:**
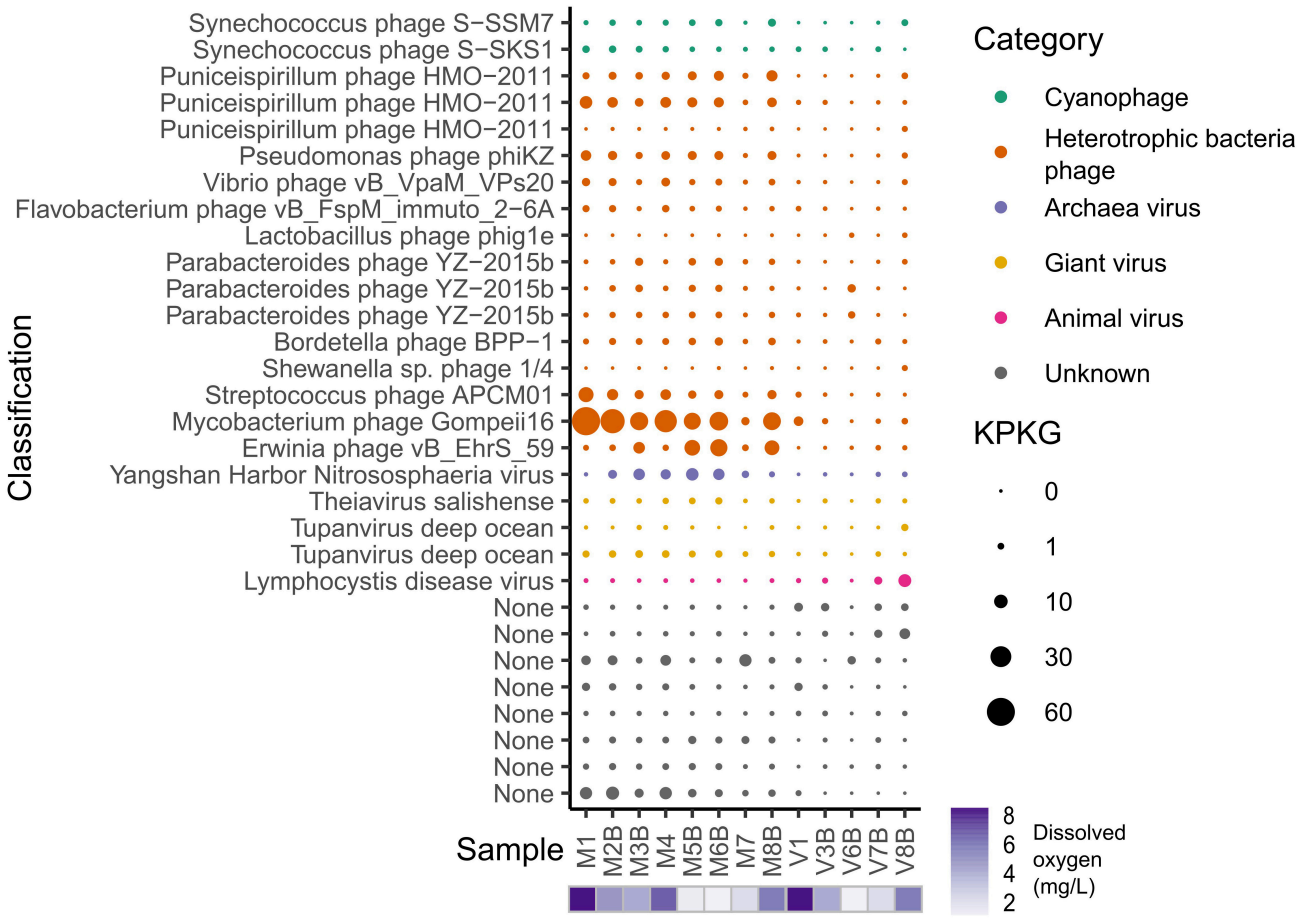
Abundance and classification of the top 30 most abundant viral populations. KPKG is total mapped nucleotides (KB) per KB of genome per GB of metagenome.

Of the top 30 most abundant viral populations, 17 were identified as bacteriophages and five as archaeal or eukaryotic viruses (Figure 3). Most of the top 30 viruses were more abundant in the microbial cellular fraction, while the Lymphocystis disease virus (a virus typically part of fish flora) and some unknown viruses were more abundant in the virion fraction. The most abundant virus was Mycobacterium phage Gompeii16, a phage isolated from soil. This may be due to the amount of soil runoff in the estuarine environment. The *Nitrososphaeria* (formerly known as *Thaumarchaeota*) virus was more abundant in samples with lower oxygen (Figure 3). Since most *Nitrososphaeria* are ammonia-oxidizing archaea (AOA), they are more tolerant of hypoxic conditions and tend to be highly abundant in low oxygen environments (Zhou et al., 2023; Ren and Wang, 2022; Hernández-Magaña and Kraft, 2024). *Nitrosopumilus* appears to be more abundant in sample 8B (Supplementary Figure S4B) which is a sample with relatively higher dissolved oxygen and salinity. Since *Nitrosopumilus* is mostly associated with pelagic environments and its high abundance was only observed in sample 8B and not the other higher dissolved oxygen samples such as sample 1–4, this may be a result of influx of oceanic water just outside of the stagnant hypoxic zone. Since viruses can only replicate in host cells with active metabolism, it is expected for *Nitrososphaeria* viruses to be more abundant in low oxygen samples. The *Synechococcus* phages and *Puniceispirillum* phages infect bacteria that tend to be widespread across marine and estuarine environments (Figure 3). However, their bacterial hosts do not appear to be particularly abundant in this study, suggesting possible high viral lytic activity (Supplementary Figure S4A).

### General AMG trends: virion fraction yields more viral populations, but less AMGs

**Figure 4:**
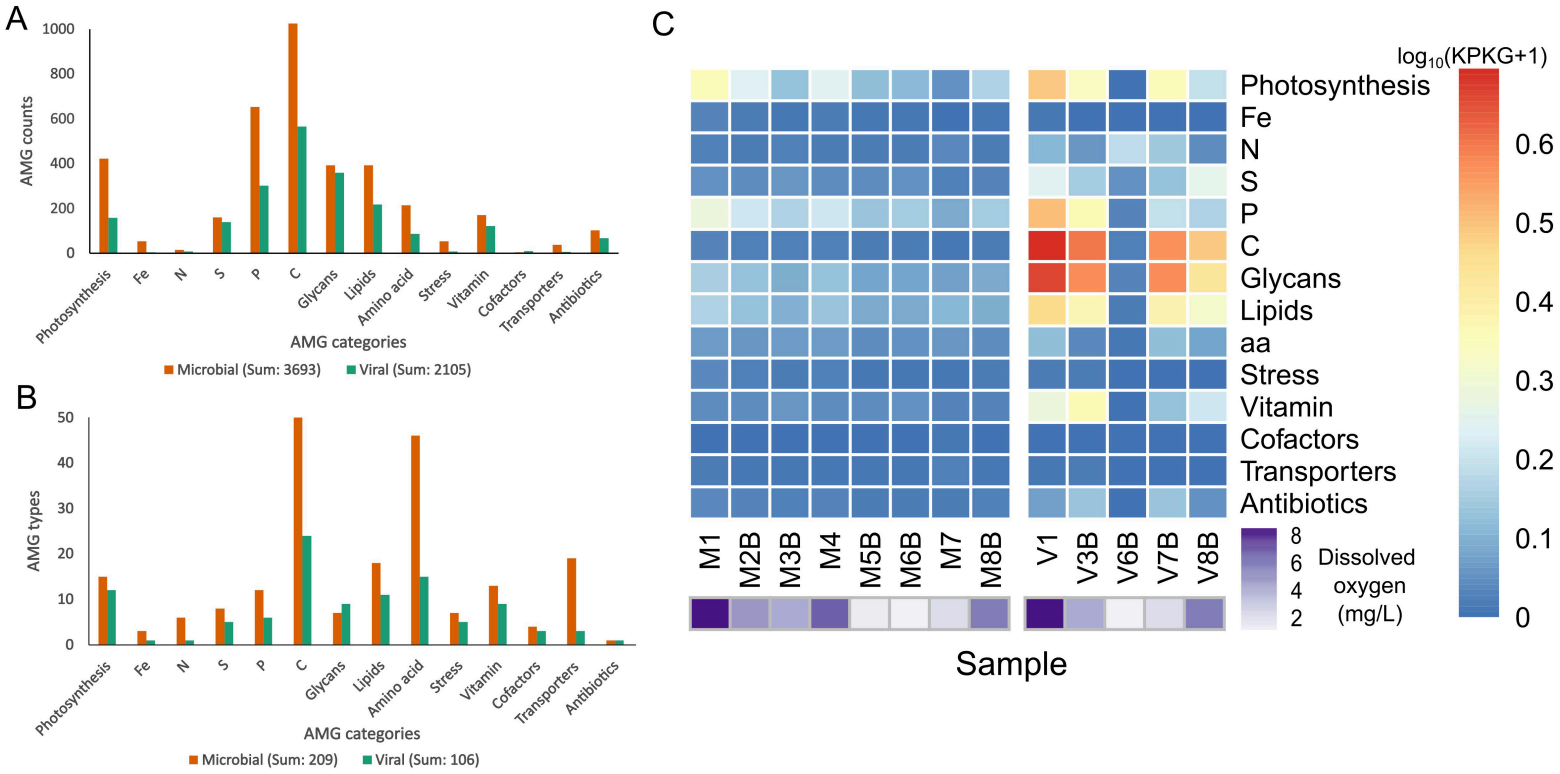
AMG categories identified from both fractions. “Microbial” refers to the 0.2–3 μm fraction and “Viral” refers to the < 0.2 μm fraction. **(A)** AMG raw counts; **(B)** non-redundant AMG types; **(C)** AMG abundance of each category in each sample. The relative abundance of the viral contig containing the AMG was used for AMG abundance estimation.

In total, 3,693 AMGs were identified from the microbial cellular fraction and 2,105 AMGs were identified from the virion fraction (Figure 4A, Supplementary Table S4). Almost twice the quantity of AMGs were identified from the microbial cellular fraction, despite yielding less overall viral populations; the AMG-per-virus ratio for microbial cellular fraction is 0.42, while it is only 0.17 the virion fraction (Table 1, Figure 4A, Supplementary Table S4). Correspondingly, the microbial cellular fraction showed twice as much diversity of AMGs compared to the virion fraction (Figure 4B).

The majority of AMGs were related to photosynthesis or carbon metabolism, and they are also the most abundant, concurring with a previous study (Figure 4, Supplementary Table S4; Hurwitz et al., 2013b). The most abundant AMGs in the microbial cellular fraction were involved in photosynthesis, while the top AMGs in the virion fraction were related to core carbon metabolism or glycan metabolism (Figure 4C). Overall, AMGs are more abundant in samples with higher oxygen levels, with sample 1 showing the highest relative abundance of AMGs in both fractions (Figure 4C). A possible explanation is relatively low cellular metabolic activity during oxygen limitation, leading to less viral replication, and thus less AMG acquisition.

In recent years, the concept of a group of globally conservative “core” AMGs across different environments has been proposed, which include the 14 genes *dcm, cysH, folE, phnP, ubiG, ubiE, waaF, moeB, ahbD, cobS, mec, queE, queD*, and *queC* (Kieft et al., 2020). These “core” AMGs are suggested to be globally conserved and perform essential viral functions regardless of host or environment. Meanwhile, most other AMGs occur more sporadically and are relatively environmentally specific. In the Baltic Sea, which is also a brackish environment, eight of these 14 AMGs were found (Heyerhoff et al., 2022). However, only three of the 14 proposed “core” AMGs were found in this study (*folE, cobS*, and *queC*), suggesting that although certain “core” AMGs persist, overall virus AMG composition show some level of adaptation to the local environment (Supplementary Table S5). Curiously, these three shared AMGs are all involved in vitamin/cofactor metabolism, implying that auxiliary vitamin/cofactor metabolism may be an important function of viral AMGs.

### Cyanophages, photosynthesis AMGs and Fe metabolism AMGs

**Figure 5:**
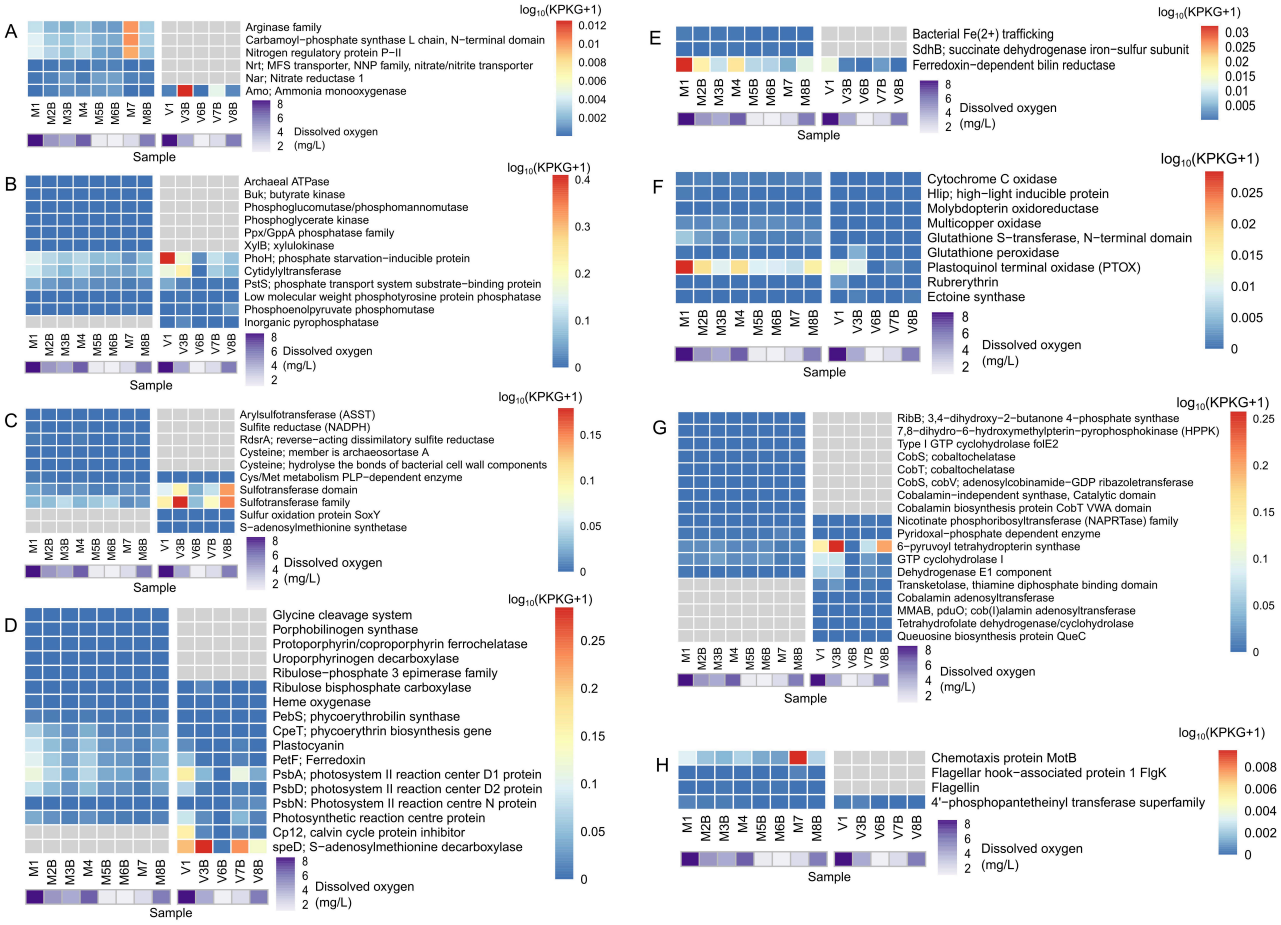
AMG abundance of different metabolic categories. The abundance of the viral contig containing the AMG was used for AMG abundance estimation. Due to the difference in magnitude across different AMG groups, separate scales and legends are used for each panel. **(A)** N metabolism; **(B)** P metabolism; **(C)** S metabolism; **(D)** photosynthesis; **(E)** Fe metabolism; **(F)** stress response AMGs; **(G)** vitamins; **(H)** cofactors.

The most well-studied AMG, the viral *psbA* gene, can consist as much as 50% of *psbA* gene expression in cyanobacteria, indicating the important contribution of viral AMGs in photosynthesis and in turn, marine microbial production (Gao et al., 2016; Sieradzki et al., 2019). The most abundant photosynthesis AMGs found in this study were *psbA, petF* ferredoxin, *speD* and *cp12* genes, which are known to be widespread in cyanophages (Figure 5D; Gao et al., 2016). PsbA is a photosystem II reaction center protein, PetF ferredoxin is a Fe-containing protein that enables electron transfer between photosystems, CP12 is a protein that regulates the carbon fixation process, and *speD* is a gene that synthesizes polyamines that regulate photoadaptation (Kotzabasis et al., 1999; Supplementary Table S6). The distribution of ferredoxin-dependent bilin reductase matched the distribution of ferredoxin, suggesting an active role of viral AMGs in light harvesting (Figures 5D, E). As a limiting nutrient in the marine environment, the presence of bacterial Fe trafficking AMGs in viruses suggests the potential assistance viruses provide to their hosts during Fe nutrient limitation (Figure 5E).

Overall, most photosynthesis AMGs were more abundant in samples with higher oxygen levels, which is consistent with the distribution of cyanobacteria and cyanophage (Figure 5D, Supplementary Figure S6). In a eutrophic freshwater lake, *psbA* AMGs were found to be absent during lower light levels, which is consistent with the current pattern (Bhattarai et al., 2025). The only cyanobacterial MAG *Synechococcus* sp. LTW-R was more abundant outside of the hypoxic zone (Supplementary Figure S6). Correspondingly, cyanophages also tend to be more abundant in samples with higher levels of dissolved oxygen (Figure 3). The relative abundance of the cyanobacterial MAG was plotted alongside that of cyanophages, showing that the variation of cyanophages across samples approximately matched that of their host (Supplementary Figure S6). The relative proportion of Synechococcus phage S-SSM7 increases further from the shore, while the proportion of Synechococcus phage S-SKS1 decreases (Supplementary Figure S6). Synechococcus phage S-SSM7 was isolated from the Sargasso Sea, while Synechococcus phage S-SKS1 was isolated from the North Sea near the coast, with these results suggesting the more “marine” profile of Synechococcus phage S-SSM7 (Sullivan et al., 2010).

While other groups of AMGs showed more patchy distribution across fractions, most photosynthesis AMGs were present in both the cellular and virion fractions, suggesting persistent participation of viral auxiliary metabolism in photosynthesis, while AMG participation in other metabolic functions is more situational. The synergy between cyanobacteria abundance, cyanophage abundance, and photosynthesis AMG abundance suggests strong metabolic ties between virus and host during photosynthesis (Figure 5D, Supplementary Figure S6).

### AMGs related to nitrogen, phosphorus and sulfur cycling

The most abundant nitrogen metabolism related AMGs were related to nitrogen excretion, carbamic acid synthesis and nitrogen availability regulation, and they were only found in the microbial cellular fraction (Figure 5A, Supplementary Table S6). The primary reaction in OMZs in the open ocean is thought to be denitrification (Zehr and Kudela, 2011). In archaea, the presence of nitrate reductase gene *nar* is more common in low oxygen zones, and is thought to enhance microbial survival via nitrate respiration when oxygen levels are low (Rinke et al., 2019). Indeed, nitrate reductase was more abundant in the hypoxic samples compared to the oxygenated samples in the estuary (Figure 5A).

Ammonia monooxygenase (*amo*) is a widespread and actively expressed AMG in the marine archaea *Nitrososphaeria*, thought to augment host ammonia oxidation (Ahlgren et al., 2019). In oceanic OMZ, the *amo* AMG is more abundant in surface waters (Gazitúa et al., 2021). In contrast, the *amo* AMG was not more abundant in surface waters in the current estuarine samples (Figure 5A). Other than *amo*, no nitrogen metabolism related AMGs were found to be present in the virion fraction.

The most abundant AMG involved in phosphate metabolism was the phosphate starvation-inducible protein (*phoH*), found in both microbial cellular and virion fractions, and more abundant in samples closer to the coast, where cyanobacteria were more abundant (Figure 5B, Supplementary Table S6). In a freshwater lake, *phoH* was observed to be more abundant before an algal bloom, showing a negative connection with cyanobacteria (Bhattarai et al., 2025). This suggests that the *phoH* AMG acquisition in freshwater and estuarine environments may follow different patterns. Despite being a AMG, *phoH* is so prevalent in marine phages that it has been proposed to use *phoH* as a signature gene for assessing marine phage diversity (Goldsmith et al., 2011). In a wetland study, the phosphorus metabolism AMGs *phoH, phoU* and *pstS* genes were prevalent in all environments and genetically diverse, but their diversity was not correlated with either habitat or host (Yu et al., 2023). The ubiquity of the *phoH* AMG in both marine and freshwater habitats indicates the importance of phage auxiliary metabolism in aquatic microbial phosphate assimilation.

*PstS*, the phosphate assimilation gene, was also more abundant closer to the coast (Figure 5B, Supplementary Table S6). Since cyanobacteria were more abundant in samples closer to the coast (Supplementary Figure S6), a possible explanation is that spring estuarine algal blooms depleted the available phosphorus in the water, thus giving *phoH* and *pstS* carrying viruses an advantage in augmenting the survival of their hosts when coping with phosphate limitation.

For other AMGs related to phosphorus metabolism, cytidylyltransferase, a gene involved in bacterial cell wall synthesis, was the most abundant AMG, and was more abundant in samples taken closer to the surface (Figure 5B, Supplementary Table S). The presence of cytidylyltransferase as a viral AMG was also observed in Lake Baikal (Coutinho et al., 2020) and found in multiple cyanophage genomes (Rong et al., 2022). It is unclear why viruses carry this AMG, but its distribution pattern and presence in cyanophage suggests it may serve some form of photoprotective function for its host.

In contrast to other groups of AMGs, some sulfur metabolism related AMGs, such as sulfotransferase, were more abundant in bottom level samples with lower oxygen, corresponding to how most marine sulfur cycling happens in and around sediments (Figure 5C, Supplementary Table S6; Wasmund et al., 2017). Sulfur metabolism AMGs are heavily present in a permanently anoxic coastal basin, suggesting the contribution of viral AMGs to sulfur cycling (Mara et al., 2020). *RdsrA* is a sulfur cycling gene that may function in either the reductive or oxidative direction. Here, it was found to be more abundant in higher oxygen environments and was only to be seen in microbial samples (Figure 5C; Carolan et al., 2015). *SoxY* was found to be unique to viral samples among type I AMGs (Figure 5C, Supplementary Table S7). *SoxY* is a common AMG carried by phages to augment the thiosulfate oxidation pathway and potentially increase energy yield toward viral replication (Supplementary Table S6; Kieft et al., 2021).

### AMGs related to stress, vitamins and cofactors

AMGs responding to environmental stress included genes that protect the cell from oxidative damage, high light, copper poisoning, phosphate limitation, and osmotic stress (Figure 5F, Supplementary Table S6), which are all common sources of stress in the estuarine environment. In the Baltic Sea redoxcline, it has been found that viruses mostly invest their resources into stress defense rather than into proliferation (Köstner et al., 2017). The variety of stress response AMGs found in this study supports the notion that viruses tend to invest in stress defense in hypoxic environments.

The plastoquinol terminal oxidase (PTOX), a gene that serves as a safety valve against photooxidative stress, was more abundant in samples closer to the surface, indicating that viral AMGs can protect their hosts from photooxidative damage (Figure 5F, Supplementary Table S6). The presence of ectoine synthase, an osmotic stress protectant, and the fact that it was not found among host-associated virus AMGs in the microbial cellular fraction, suggests that viruses may help their hosts adapt to the variable salinity of the estuarine environment (Figure 5F, Supplementary Table S6).

The vitamin metabolism AMGs observed were relatively more abundant in the virion fraction compared to the microbial cellular fraction, which mirrors previous observations that vitamin AMGs are significantly more enriched in free living microbial samples compared to particle associated samples (Coutinho et al., 2023; Figure 5G). Curiously, only genes related to B family vitamins were found, including vitamin B1 (thiamin), vitamin B2, vitamin B3, vitamin B6, vitamin B9 (folate) and vitamin B12 (cobalamin; Figure 5G, Supplementary Table S6). Although B family vitamins play a central metabolic role in the marine environment, large portions of the ocean are devoid of it (Sañudo-Wilhelmy et al., 2014).

The two most prominent processes these vitamin AMGs are involved in are folate and cobalamin metabolism. For folate metabolism, the most abundant vitamin AMG was 6-pyruvoyl tetrahydropterin synthase (PTPS), a gene that facilitates folate biosynthesis, found to be significantly more abundant in the virion fraction (Figure 5G, Supplementary Table S6). Viral AMGs encoding folate synthesis were found to be abundant in macroalgae associated bacteria, suggesting the supporting role of viruses in marine folate production (Zhao et al., 2024). Folate AMGs were also found to be abundant in the dispersed virus community in deep water, with one virion encoding five genes in the folate decomposition pathway, indicating that viruses may carry AMGs to either synthesize or utilize folate depending on environmental conditions (Coutinho et al., 2023). However, it should be noted that the folate may be used for *de novo* nucleotide synthesis, thus possibly serving the virus's own replication needs instead of being of auxiliary function to the host (Martin et al., 2025). As for cobalamin metabolism, AMGs facilitating cobalamin biosynthesis were found to be common in both fractions in the estuarine hypoxic zone; *cobT* was also detected in the Pacific OMZ (Jurgensen et al., 2022). Cobalamin production is complex and energy expensive, thus cobalamin is synthesized by relatively few groups of prokaryotes in the marine environment, with some bacteria able to salvage cobamide precursors (Soto et al., 2023; Shelton et al., 2019). The prevalence of cobalamin synthesis viral AMGs may provide their hosts a survival advantage during cobalamin limitation. Curiously, no cobalamin related AMGs were shared between the fractions; the AMGs in the bacterial fraction were all concerned with aerobic synthesis of cobalamin or cobalamin-independent synthesis of methionine, while the AMGs in the virion fraction facilitate the conversion of cobabalmin to coenzymes (Figure 5G, Supplementary Table S6). While none of these cobalamin related genes were particularly abundant, this pattern hints at the different metabolic roles of host-associated viruses and planktonic viruses regarding sought-after vitamins.

Notably, several flagellar structure AMGs were present in the microbial cellular fraction (Figure 5H, Supplementary Table S6). An AMG encoding for flagellar formation was previously observed in a roseophage (Huang et al., 2021). Flagellar AMGs were also found to be present in bacteriophages in glacier ice and nanoarchaea viruses in a chemotrophic-based underground estuary (Zhong et al., 2021; Ghaly et al., 2024). Since the flagellar filament is a phage receptor, the presence of flagellar AMGs may make their host more susceptible to phage infection (Gonzalez et al., 2018). Alternatively, their presence may also help to enhance the mobility and thus survival of their hosts, since the most abundant AMGs was of the rotary motor and not of the flagellar filament (Figure 5H, Supplementary Table S6). More experimental validation is needed to understand the role of viral auxiliary metabolism in cellular flagellar formation.

### Type I AMGs from the microbial cellular fraction show more functional diversity

Comparison of shared gene content between microbial genes and viral genes showed that 16% of microbial genes were shared between the virion fraction and non-viral microbial bins (Supplementary Figure S7A). The above set of shared gene content consists 73% of viral genes, showing that most viral genes can also be found among microbial bins. Since viral content was discarded from the microbial cellular fraction for this analysis, this indicates significant metabolic overlap between the virus and cellular microbial genomes.

Of the 106 identified AMGs with KO entries (type I AMGs), 81 could only be found in viruses derived from the microbial cellular fraction, eight were unique to the virion fraction, and 17 could be found in both (Supplementary Figure S7B). This indicates that viruses associated with the microbial cellular fraction carry type I AMGs that can handle more auxiliary metabolic functions, suggesting that host-associated viruses may have a wider range of metabolic capability compared to dispersed viruses. These results are consistent with overall AMG identification results, which show more AMGs in the microbial cellular fraction than in the virion fraction (Figures 4A, B, Supplementary Table S4). Another possible explanation is potential active genetic exchange between viruses and their hosts, resulting in increased amounts of shared metabolic genes. Meanwhile, most of the eight AMGs unique to the virion fraction were core carbon metabolism genes, suggesting that dispersed viruses may serve an important function in core carbon metabolism, either enhancing their hosts' chance of survival, or increasing carbon energy production for their own benefit (Supplementary Table S7).

The results of RDA analysis show that statistically, very little of AMG relative abundance variance is explained by environmental factors (Supplementary Figure S8). Nevertheless, RDA visualization results suggest that photosynthesis-related AMGs are positively correlated with DO concentrations, while sulfur AMGs are negatively associated with oxygen levels. Positive correlations of photosynthetic AMGs with dissolved oxygen levels is consistent with observations in marine oxygen minimum zones, while in the same study sulfur AMGs were found to be unrelated to dissolved oxygen levels (Jurgensen et al., 2022).

### Function appears to outweigh host taxonomy for viral AMG retention

Hosts were successfully predicted for 4,265 of the 19,370 viral populations. On the phylum level, most predicted hosts were *Bacteroidota* and *Proteobacteria* (Supplementary Figure S9). Of the virus-host predictions, 241 (5.6% of predictions) derived their best hit from the native microbial MAGs, while the rest matched to hosts in the iPHoP database. In these 241 local virus-host pairs, the most common host phyla were *Bacteroidota* and *Pseudomonadota* (Supplementary Figure S10). A *Thermoplasmatota* MAG classified as MGIIa-L2 recruited 43 viruses, which was the highest number of recruited viruses out of all the local host MAGs (Supplementary Figure S10). These results may be caused by the low amount of marine group II (MGII) archaea in the iPHoP database, compared to the high abundance of MGII archaea in the PRE, resulting in many viruses recruiting to the limited amount of MGII in the database (Xie et al., 2018).

**Figure 6:**
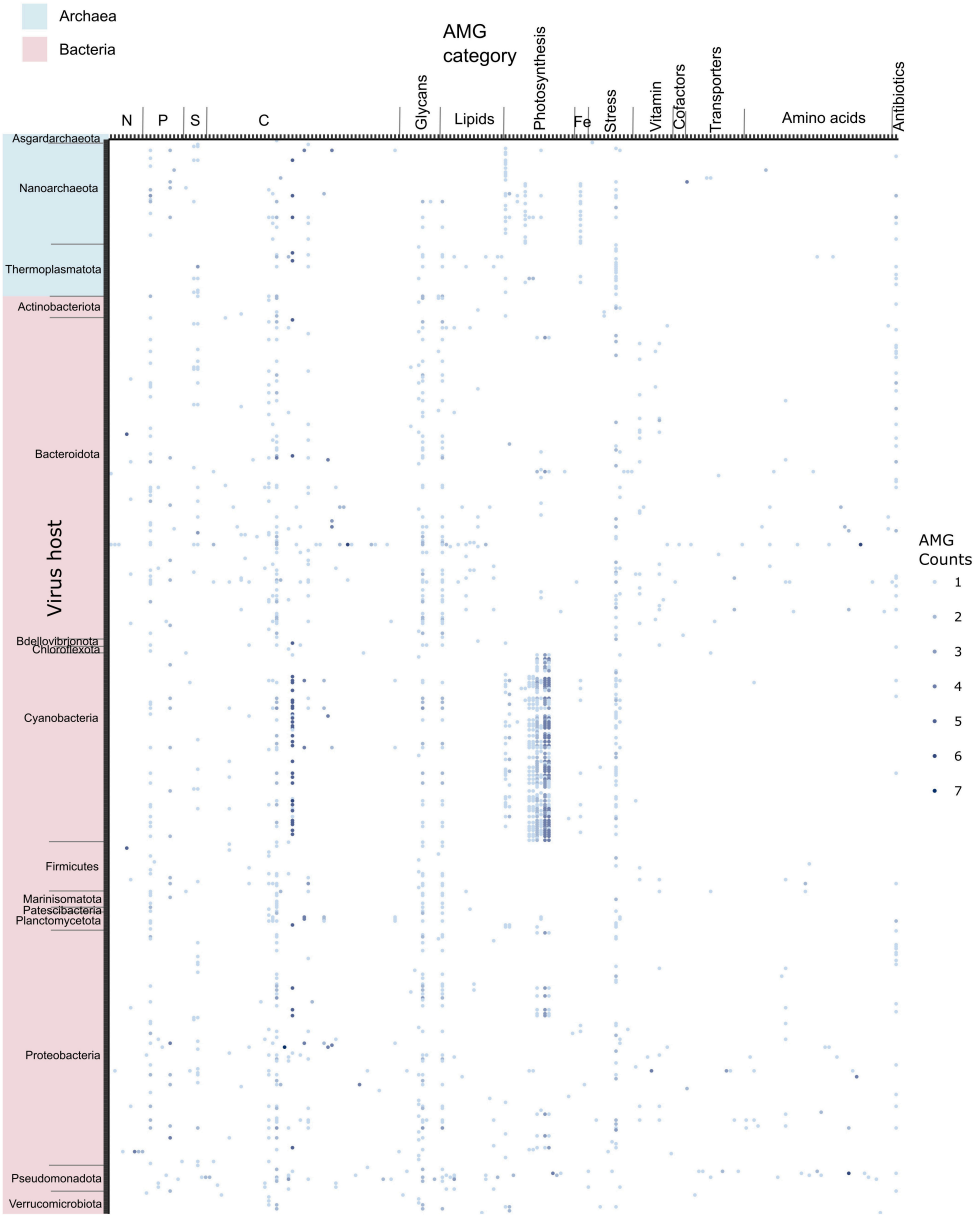
Dotplot of the 545 viruses with both host assignment and also containing AMGs. Each dot represents one virus, the color depth corresponds to the number of AMGs on each viral contig.

To search for connections between the hosts that viruses infected and the AMGs that they carried, 545 viruses with both a host prediction and AMGs were counted and visualized (Figure 6). To assess the significance of host and AMG category in explaining the variation of virus AMG presence, PERMANOVA was chosen as a statistical test since the data is zero-inflated and does not fulfill the normality assumption. The *R*^2^ values represent the relative significance of virus host and AMG category in explaining the presence or absence of virus AMGs on each virus. The results reveal that AMG category (*R*^2^ = 23%, *p* < 0.001) explained a much larger proportion of the variance compared to predicted host phylum (*R*^2^ = 7%, *p* < 0.001; Supplementary Table S8). AMG function is a more significant predictor of virus AMG retention than host assignment, supporting the notion that on an environmental scale, microbial function is more important than microbial taxonomy (Figure 6, Supplementary Table S8). It has been proposed that AMGs are sporadically acquired by virus genomes from their hosts, but due to limited genomic space, only the AMGs contributing to viral fitness are selected for, and thus persist in viruses (Zimmerman et al., 2020). Our studies support the view that the retention of AMGs in viral genomes is not random; its function is a contributing factor. The dynamic estuarine environment and stressful conditions of the hypoxic zone may exacerbate this kind of environmental selection. Nonethless, there is potential bias resulting from the fact that only a small proportion of viruses yielding both AMGs and host predictions (545 out of 19,370) were involved in this analysis.

AMGs related to nutrient metabolism were ubiquitous across viruses infecting different hosts, while AMGs related to vitamins, transporters and amino acids were less prevalent and more sporadic (Figure 6).It has been hypothesized that among cyanomyoviruses, frequently occurring AMGs are maintained through vertical inheritance, while more sporadic AMGs are subject to horizontal transferring (Crummett et al., 2016). If this observation of data is considered in the context of “core” and environmentally specific AMGs, it suggests that nutrient metabolism AMGs are more important for essential virus function, while vitamin, transporter and amino acid metabolism AMGs are more contextual to the environmental conditions. This supports the view that virus AMGs play a consistent role in microbial metabolism in the aquatic environment that contributes to overall nutrient cycling. The dynamic nature of estuarine hypoxic zone formation may give rise to more sporadic AMGs. This is supported by the fact that relatively few previously proposed “core” AMGs were found in this study (Supplementary Table S5).

There does not appear to be a significant divergence between AMGs of bacteriophages and archaeal viruses (Figure 6). The most prevalent AMGs were photosynthesis and carbon metabolism related AMGs in cyanophages, supporting the notion that cyanophages are some of the most active utilizers of AMGs in the ocean (Puxty et al., 2016; Hurwitz and U'Ren, 2016; Figure 6). Curiously, the presence of photosynthetic genes is not uncommon in the viruses infecting heterotrophic bacteria and archaea, suggesting viruses may serve some level of auxiliary autotrophic functions in marine heterotrophic microbes. Although host mispredictions are possible, the presence of photosynthetic AMGs may also be indicative of horizontal gene transfer activity by broad host range viruses. Since photosynthetic AMGs appear to be metabolically important in general, viruses carrying photosynthetic AMGs may act as genetic reservoirs that offer an advantage to host survival in energy limited conditions.

## Conclusions

This metagenomic study reveals functionally diverse viral-encoded AMGs in an estuarine hypoxic zone. Viruses derived from the virion fraction and the cellular fraction were distinct, with the cellular fraction generating fewer unique viruses, but more types of AMGs. Cyanobacteria variation, cyanophage variation, photosynthesis and Fe metabolism AMG variation were in sync with each other, supporting the notion that cyanophage and photosynthetic AMGs play a core function in the estuarine environment. Overall, AMGs appeared to be more abundant in samples with higher DO levels, indicating that viruses did not acquire more AMGs in response to hypoxic conditions. However, only three of the 14 proposed “core” AMGs were found in the current dataset, suggesting that virus AMG retention show some level of adaptation to different environments. Virus-encoded AMGs in and around the hypoxic zone were often found to have functions that assisted with nutrient limitation such as shortage of P, Fe and B family vitamins. For viruses, the functionality of their AMGs was a better predictor of their distribution than the hosts they infect, supporting the view that from an ecological standpoint, microbial function is more important than microbial taxonomy. In summary, AMG composition of estuarine viruses closely reflects the estuarine environment, can potentially assist their microbial hosts to adapt to the complex and ever-changing physicochemical conditions, and may enhance the survivability of the hosts. While more experimental validation is required to confirm the metabolic activity of these AMGs and their ecological role in estuarine hypoxia, this study provides insights into the potential interactions of viruses with their microbial hosts in stress adaptation in a coastal environment with substantial anthropological impacts.

## Data Availability

Raw reads generated in this study have been deposited in the National Center for Biotechnology Information BioProject database with the project ID PRJNA1243077. Microbial metagenome-assembled genomes (MAGs) bins (10.6084/m9.figshare.28740248, https://figshare.com/s/72c9758d779f745bc5e4) and viral populations (10.6084/m9.figshare.28740188, https://figshare.com/s/413254d138c78265cec4) have been deposited on the Figshare website under the project “Microbiome of the Pearl River Estuary summer hypoxic zone”. Raw sequences for this study have also been deposited in the BMDC National Omics Data Encyclopedia (NODE) under project ID OEP00001662 (https://www.biosino.org/node/project/detail/OEP00001662), with microbial cellular fraction under Experiment ID OEX00031539 and viral fraction under Experiment ID OEX00031538. Assembled sequences are available as analyses including microbial bins (Analysis ID: OEZ00021845) and viral populations (Analysis ID: OEZ00021846). The quality summary of viral populations is available as Table S9. Amino acid sequences of AMGs are available as supplementary files (Data Sheet 3, 4).
