# Supplementary material for: Virus-encoded metabolism may support environmental stress adaptation of microbial hosts in an estuarine hypoxic zone
Source: Front Microbiol. 2026 Mar 24;17:1785655. doi: 10.3389/fmicb.2026.1785655 (PMC13079714; doi:10.3389/fmicb.2026.1785655)
Supplement: Supplementary file 2 [file Data_Sheet_1.docx]

**Supplementary Methods**

**Statement of sampling representativity during sample collection**

A low-oxygen zone in the bottom water has tendency to appear off the western coast of the Pearl River Estuary (PRE) during the summer months [1]. The China Sea Multi-Scale Ocean Modeling System (CMOMS) was observed daily and the sampling location was determined after at least 7 days of no precipitation and 3 days of stable hypoxic zone formation [2]. September 2, 2022 was determined to be an appropriate day for collection of hypoxic zone samples. On the day, the precise sampling coordinates determined dynamically by taking exploratory samples with the purpose of locating the inner and outer edges of the hypoxic zone, to form a transect. The resulting dissolved oxygen measurements indicate that the hypoxic zone was successfully captured.

Due to the need to dynamically locate the edges of the hypoxic zone to form the transect, the precise sampling sites could not be planned ahead of time and a small, agile boat was required for sample collection. Thus a limited amount of samples were able to be collected, and appropriate conditions for RNA (transcriptomic) sample collection was not able to be achieved.

**References**

1. Zhang Z, Wang B, Li S, Huang J, Hu J. On the Intra-annual Variation of Dissolved Oxygen Dynamics and Hypoxia Development in the Pearl River Estuary. *Estuaries and Coasts* 2022; **45**: 1305–1323.

2. Gan J, Liu Z, Liang L. Numerical modeling of intrinsically and extrinsically forced seasonal circulation in the China Seas: A kinematic study. *J Geophys Res Ocean* 2016; **121**: 4697–4715.

**Supplementary figures and legends**

**
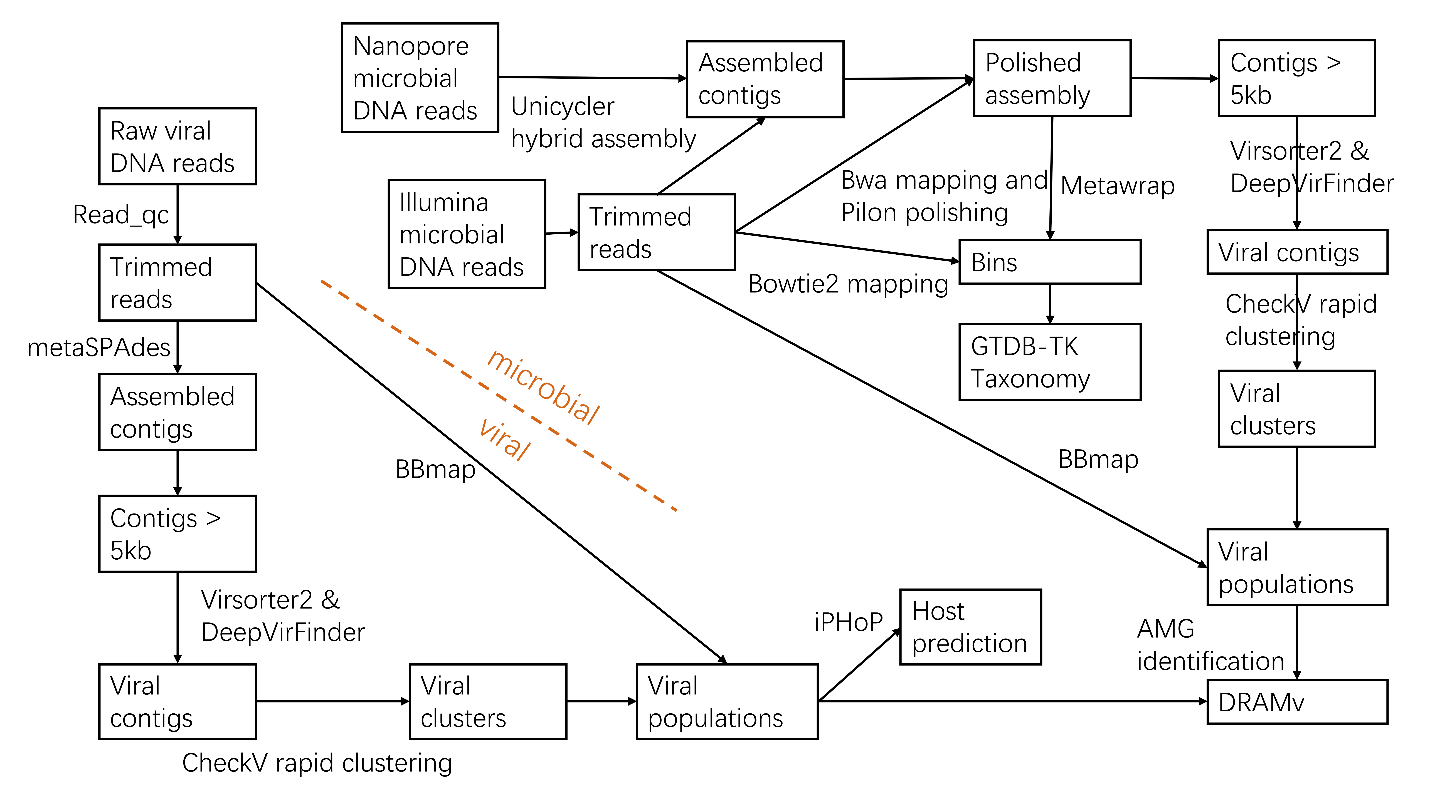
**

**Figure S1** Sequence processing method summary. Sequences from the 0.2 - 3 μm fraction are designated “microbial”, while sequences from the < 0.2 μm fraction are designated “viral”.


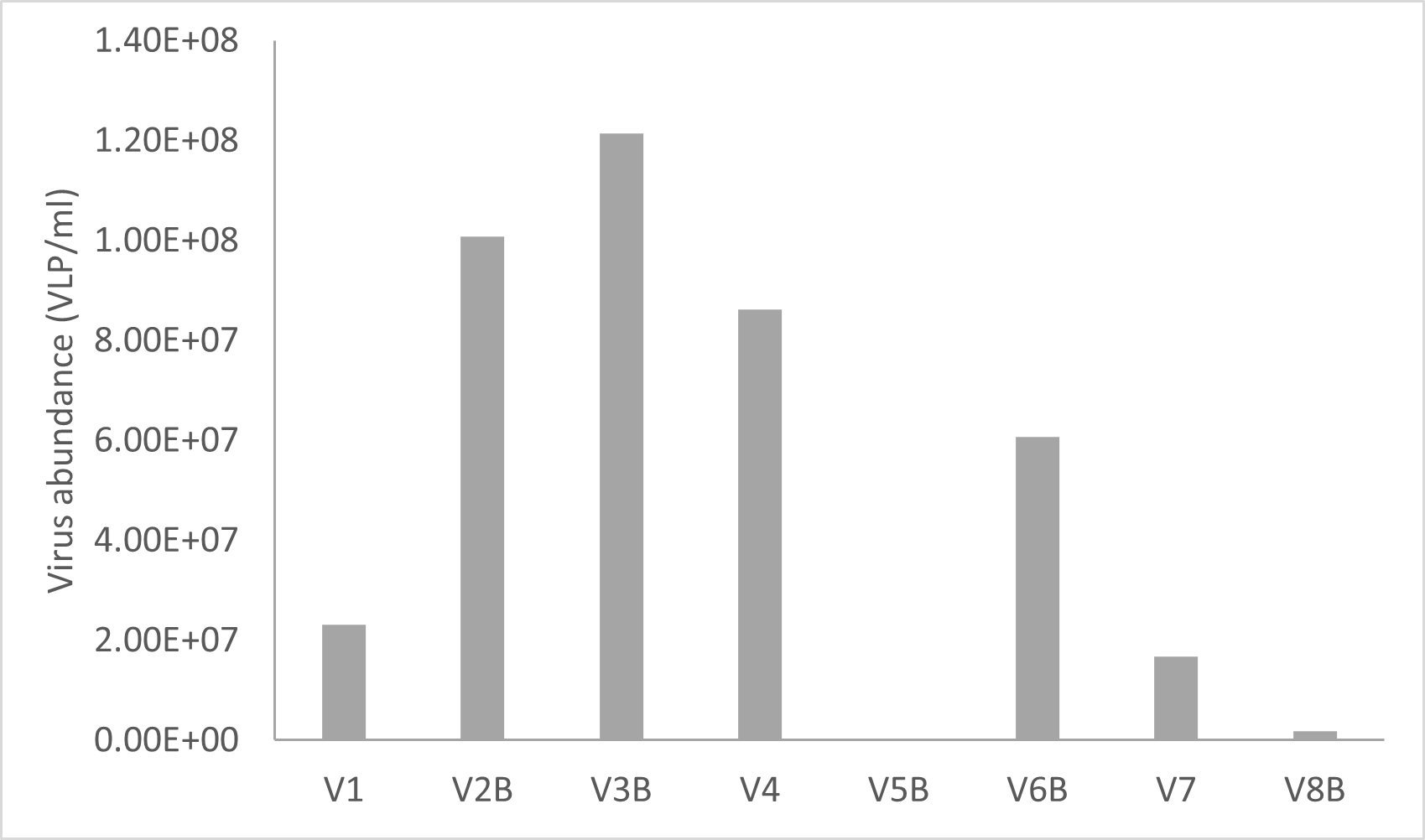


**Figure S2** Virus counts from epifluorescent microscopy. No virus particles were observed in V5B.


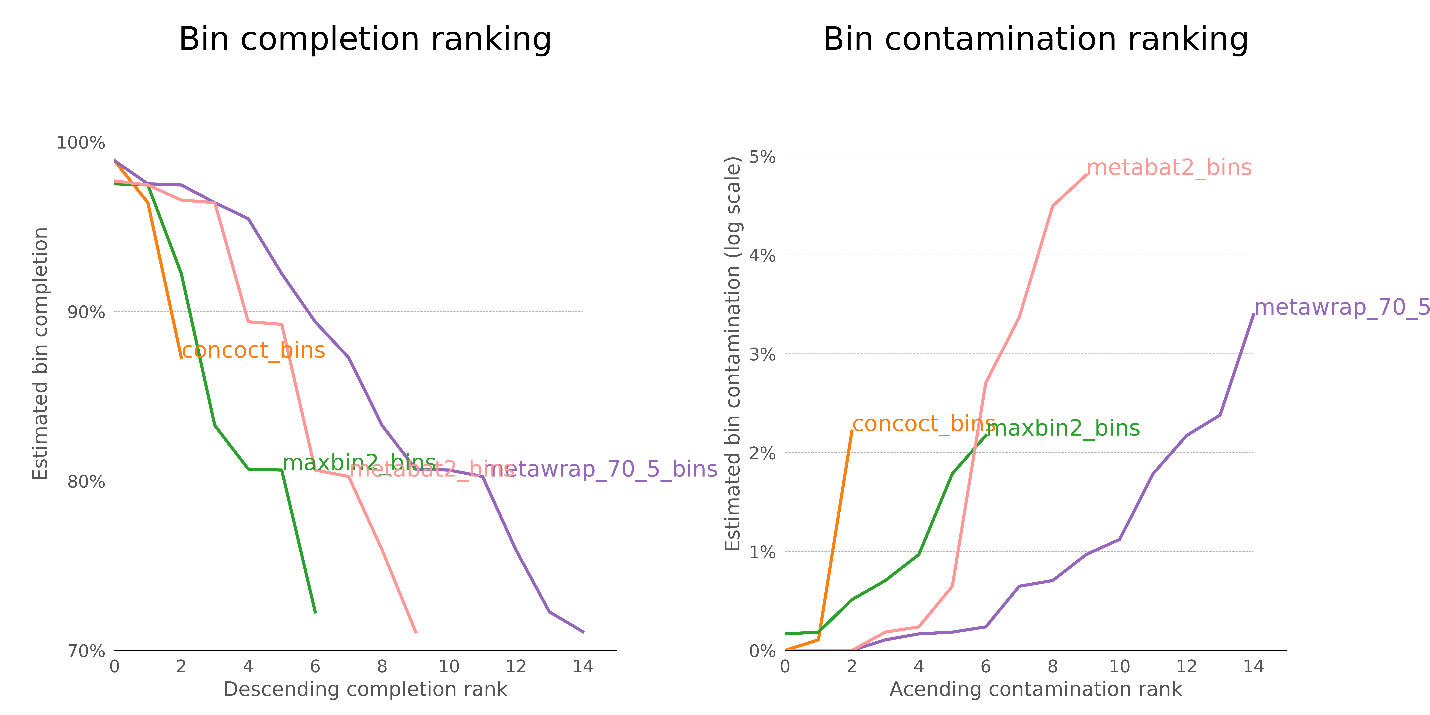


**Figure S3** Bin completion ranking and bin contamination ranking results of metagenome-assembled genome (MAG) bin refinement.


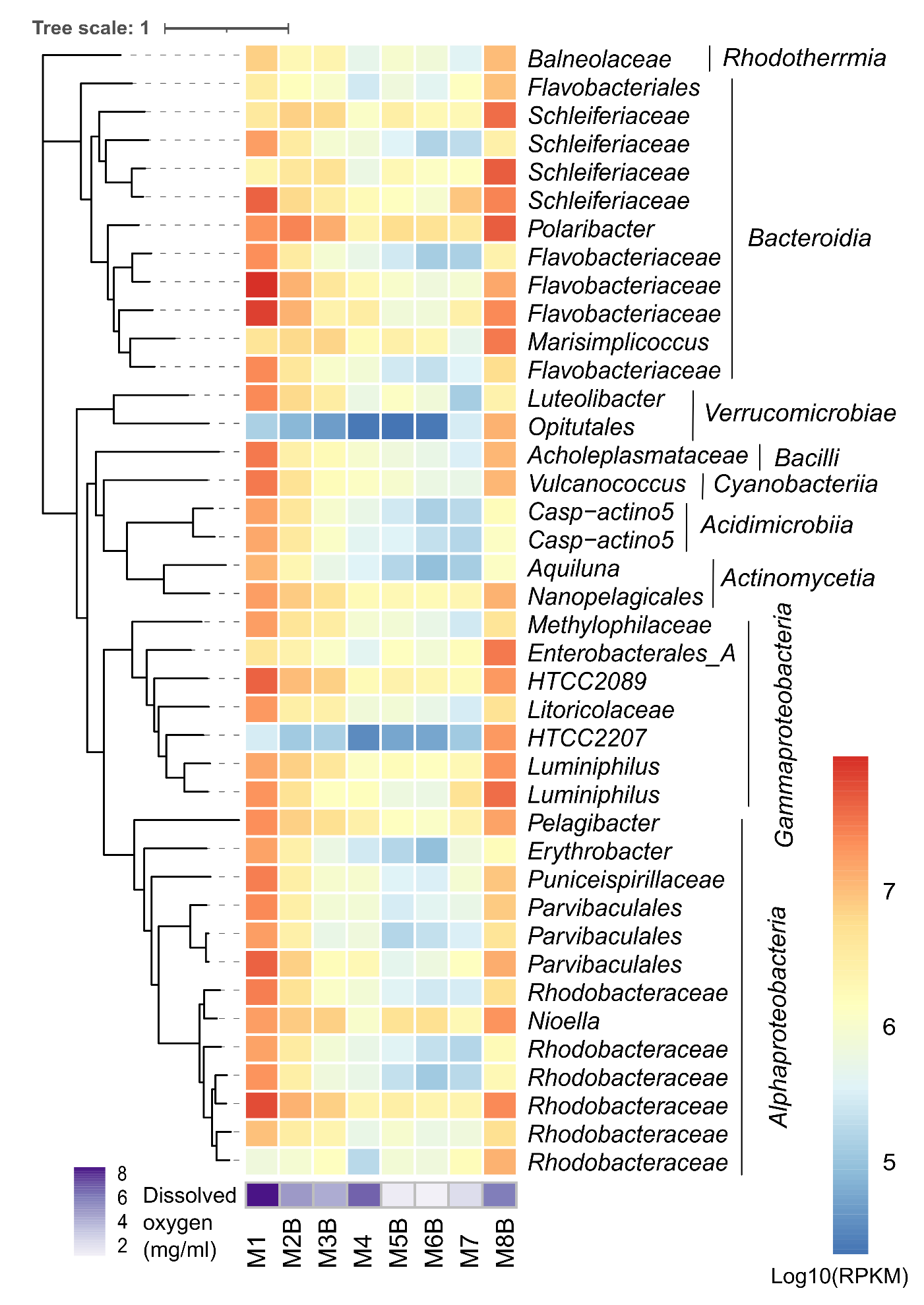


A


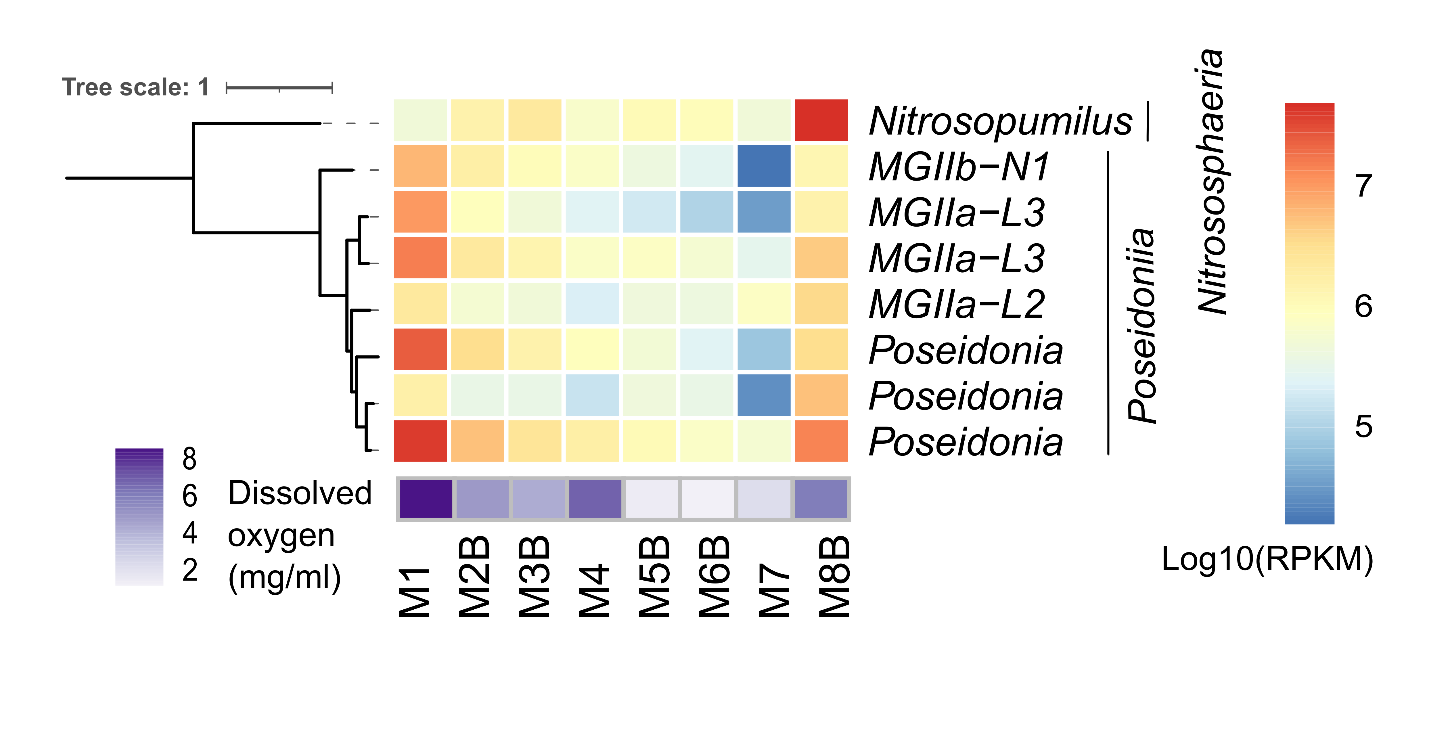


B

**Figure S4** Microbial bins abundance. Phylogenetic trees were generated using the alignments from GTDB-TK and visualized using IQ-TREE using the default parameters. (A) Top 40 most abundant bacterial MAGs. (B) All archaea MAGs.


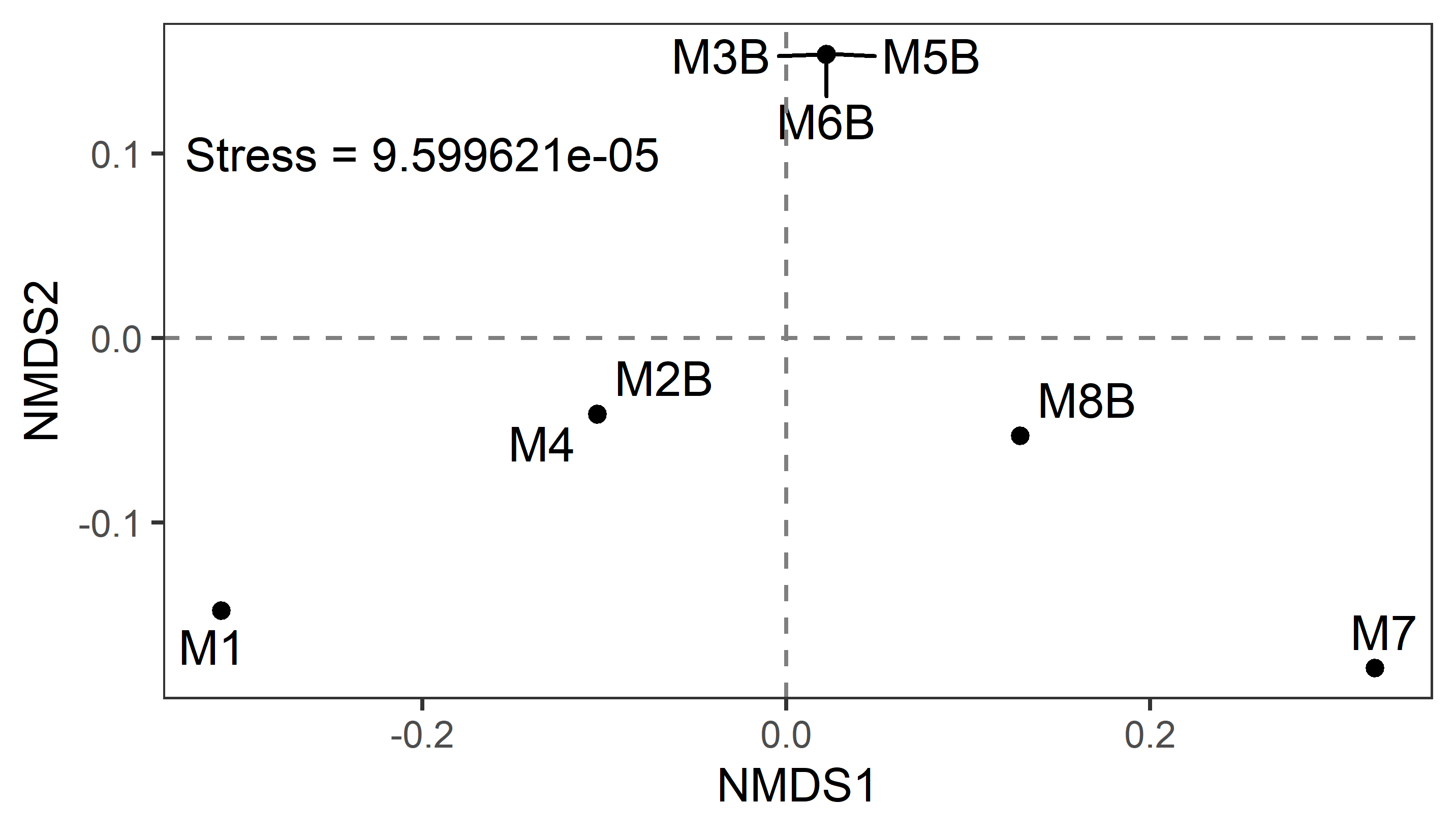

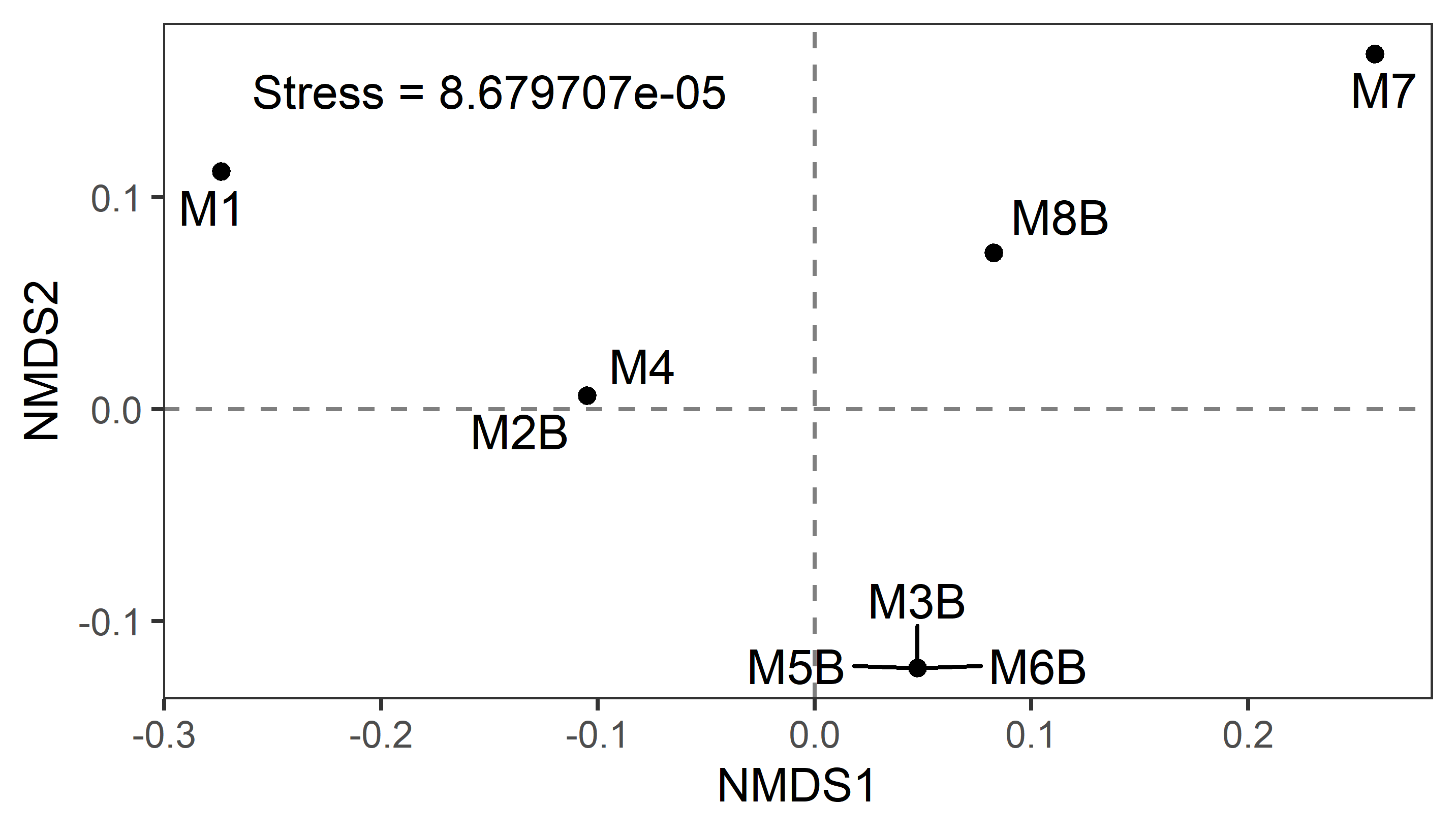

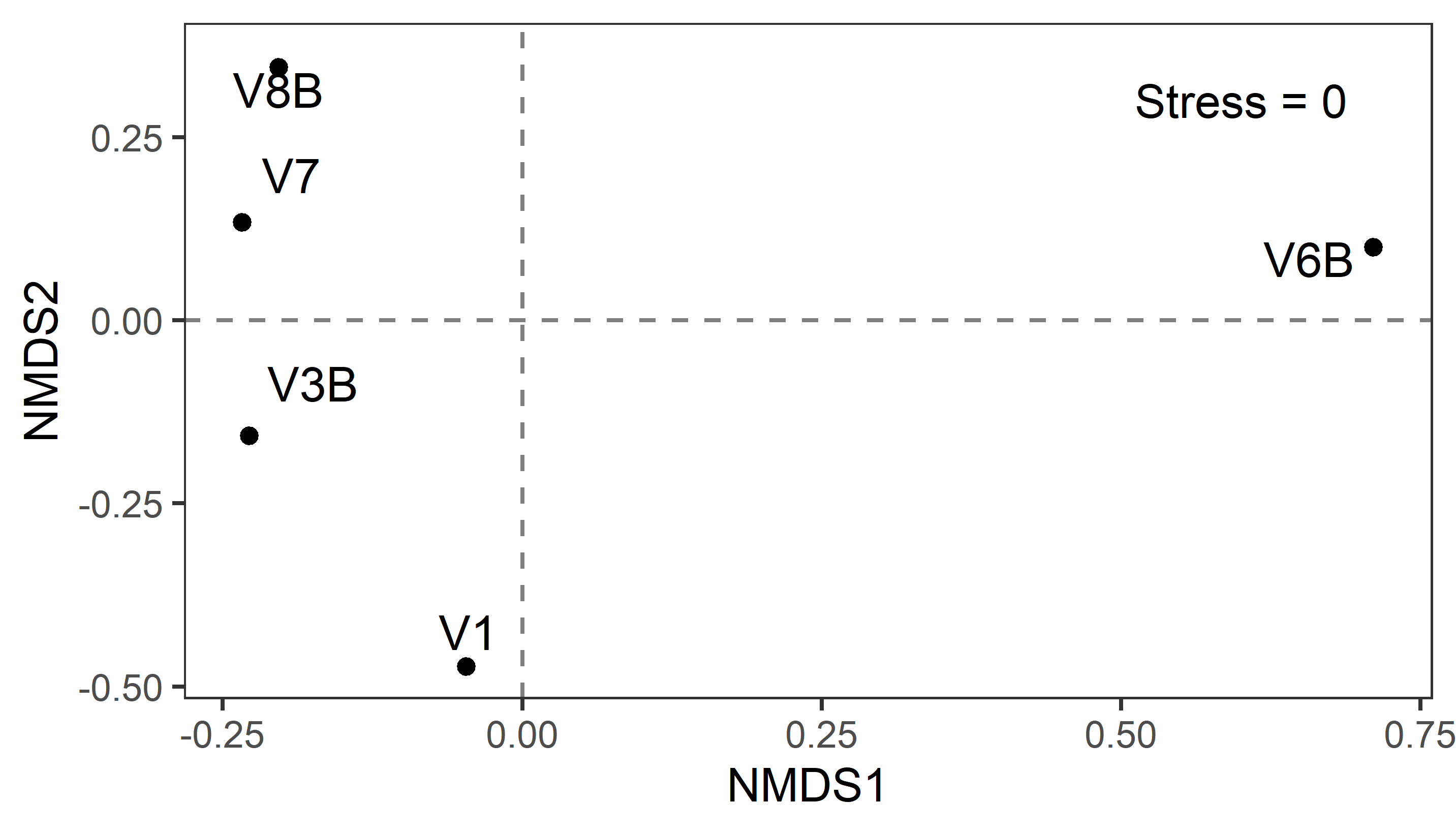


C

A

B

B


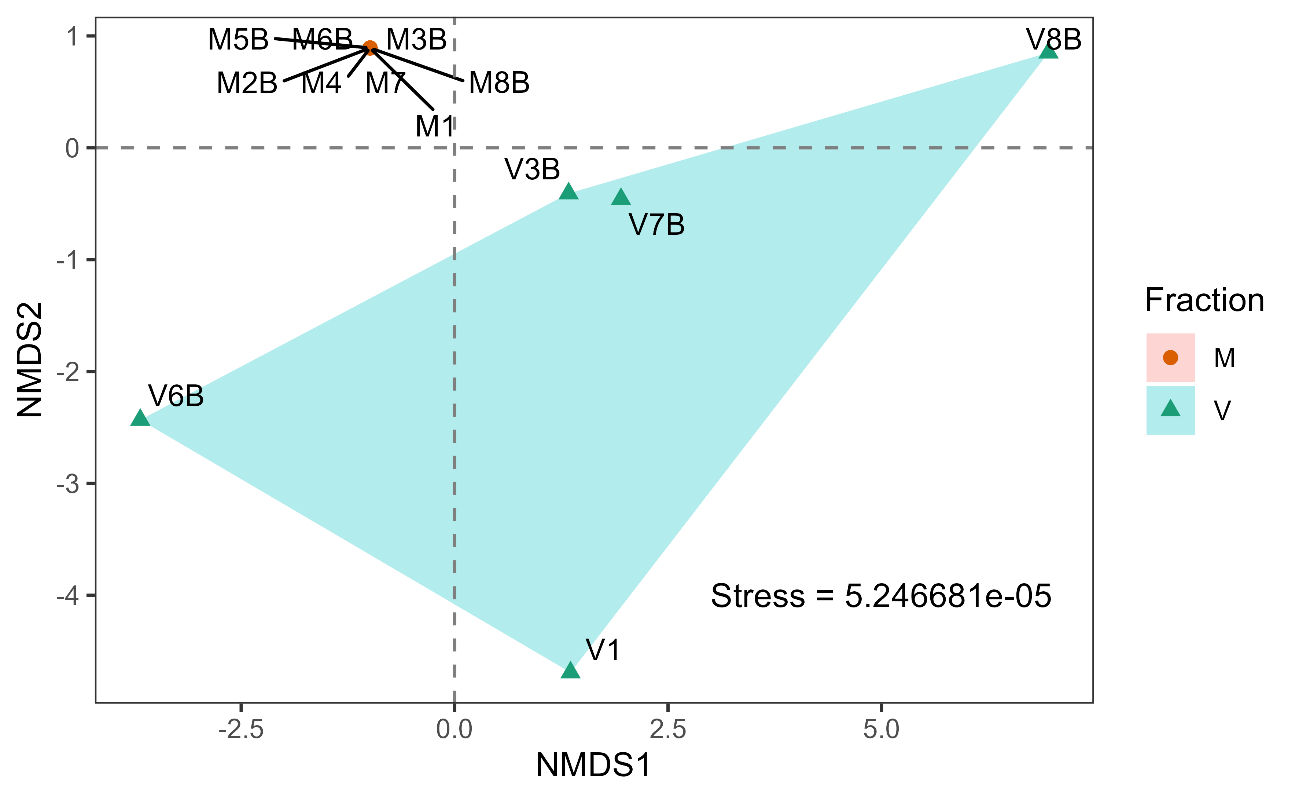


D

**Figure S5** Nonmetric multidimensional scaling (NMDS) plot of the 8 samples based on different measures of microbial and viral abundance. Due to the constraints of computational power, only the most abundant 5,000 viral populations were used in this analysis. For microbial samples, two versions of the plot were generated: using the microbial reads mapped against microbial bins, and microbial reads mapped against the top 5000 viral populations, in order to evaluate the similarity based on viral content in microbial samples. For viral community variation, the plot was generated using the results of viral reads mapped against the top 5000 viral populations. In addition, the combination of (B) and (C) were visualized in a single diagram in (D). Stress level is indicated. (A) Microbial reads mapped against microbial bins; (B) Microbial reads mapped against top 5,000 viral populations; (C) Viral reads mapped against top viral populations. (D) Combined viral and microbial visualization with microbial and viral reads mapped against top 5,000 viral populations. Orange represents the microbial cellular fraction and green represents the viral fraction.


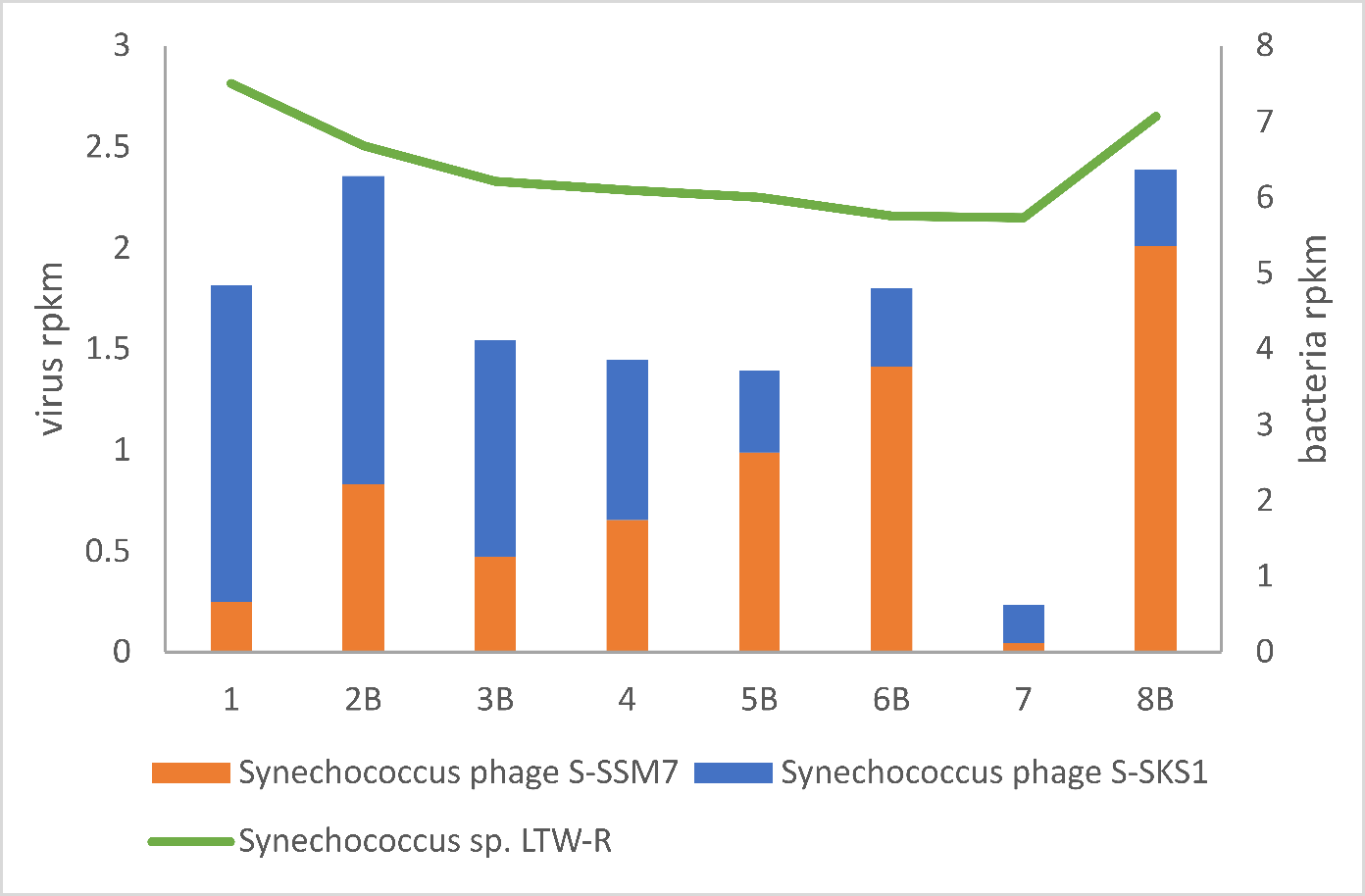


A

**Figure S6** Visualization of cyanobacteria abundance, cyanophage abundance, and photosynthesis AMG abundance. (A) Relative abundance of cyanobacteria MAGs and cyanophages identified within the top 30 viruses. A double axis plot of cyanobacteria and cyanophage rpkm is used to visualize potential co-variation. Lines represent cyanobacteria abundance and bars represent cyanophage abundance. (B) Relative abundances of photosynthesis related AMGs.


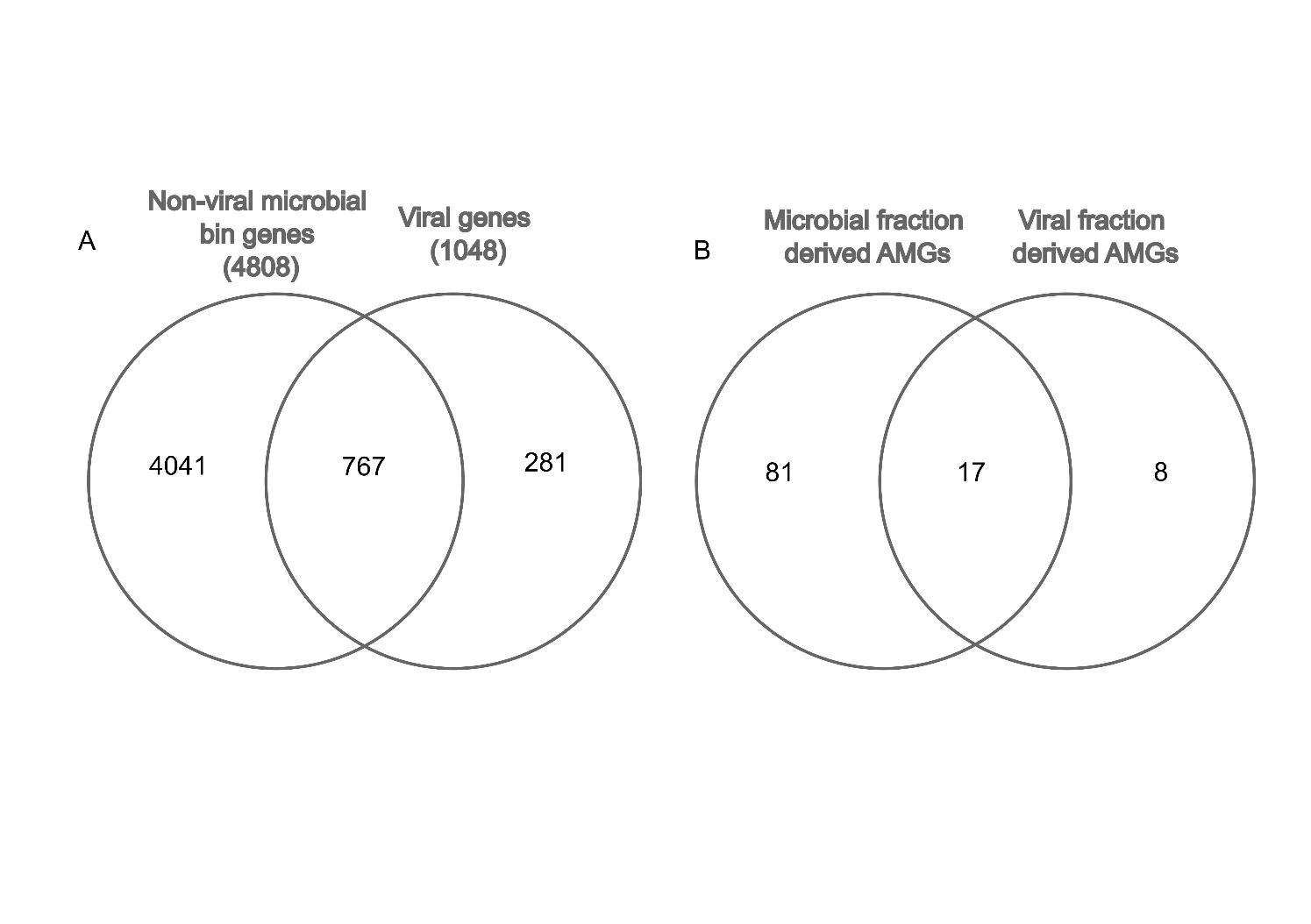


**Figure S7** Shared metabolic gene content between microbial and viral community based on KEGG Orthology (KO). To characterize the shared metabolic gene content between viral and microbial host communities, KO entries of DRAM annotated genes from the microbial bins, viral populations and identified AMGs were counted and visualized using a venn diagram. To better understand the metabolic interconnection between the viral and cellular fractions, redundant viruses present in the cellular fraction must be removed. Viral content from the cellular fraction was identified using the same Virsorter2 and DeepVirFinder method described in the methods section and removed to avoid redundancy. (A) All metabolic genes; (B) Origin of type I AMGs.


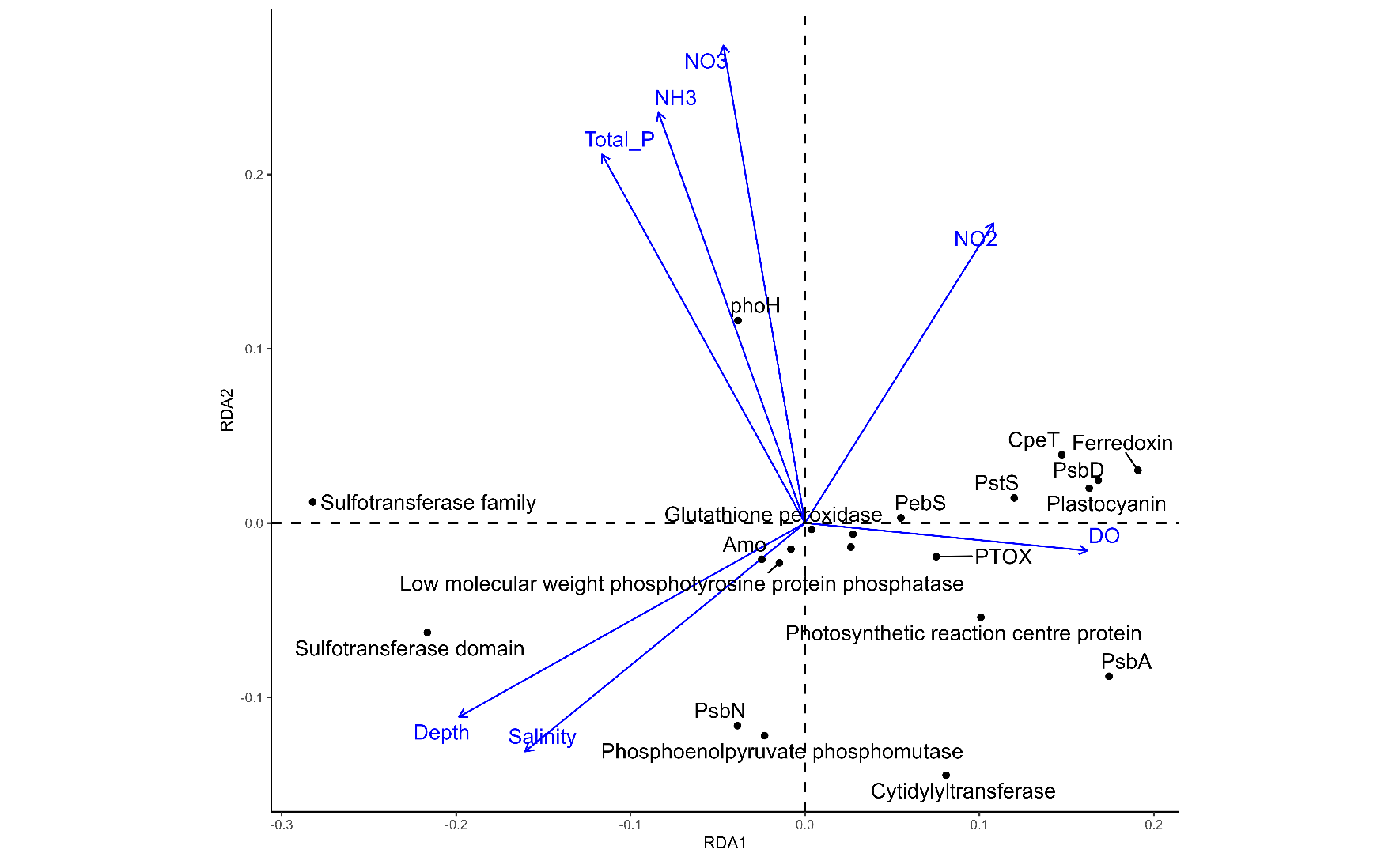


**Figure S8** Redundancy analysis (RDA) biplot of AMG relative abundance and environmental factors. RDA1 explains 3.35% of variance, while RDA2 explains 0.88% of variance. Total constrained variance is 5.17%, while total unconstrained variance is 8.31%. Each black dot represents the variation of the AMG among the samples. The angles between AMGs and environmental factors denote their degree of correlation.


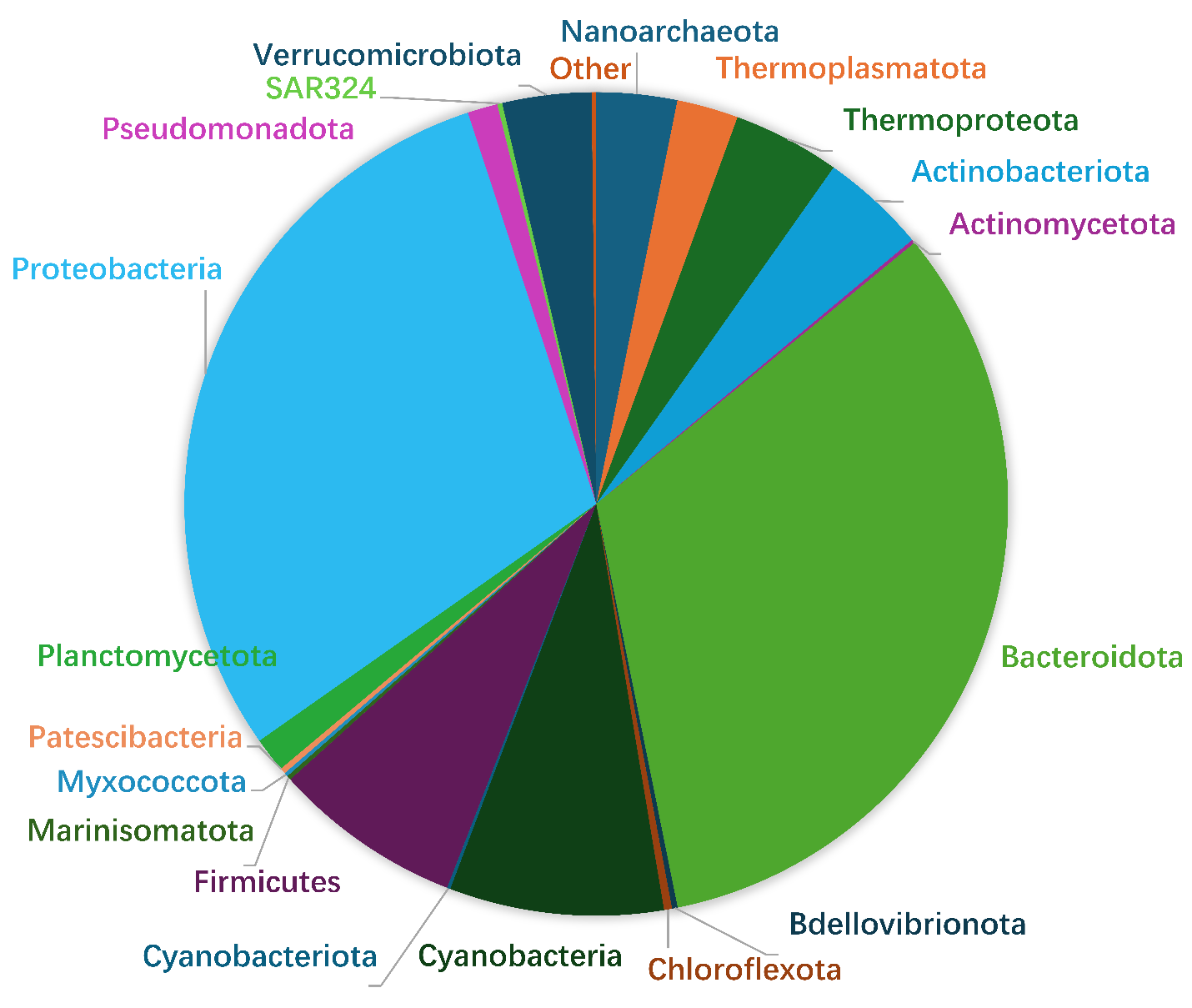


**Figure S9** Host predictions of virus populations (phylum-level taxonomy). The partitions of the pie chart show the proportions of predicted host relative to all the predicted hosts.


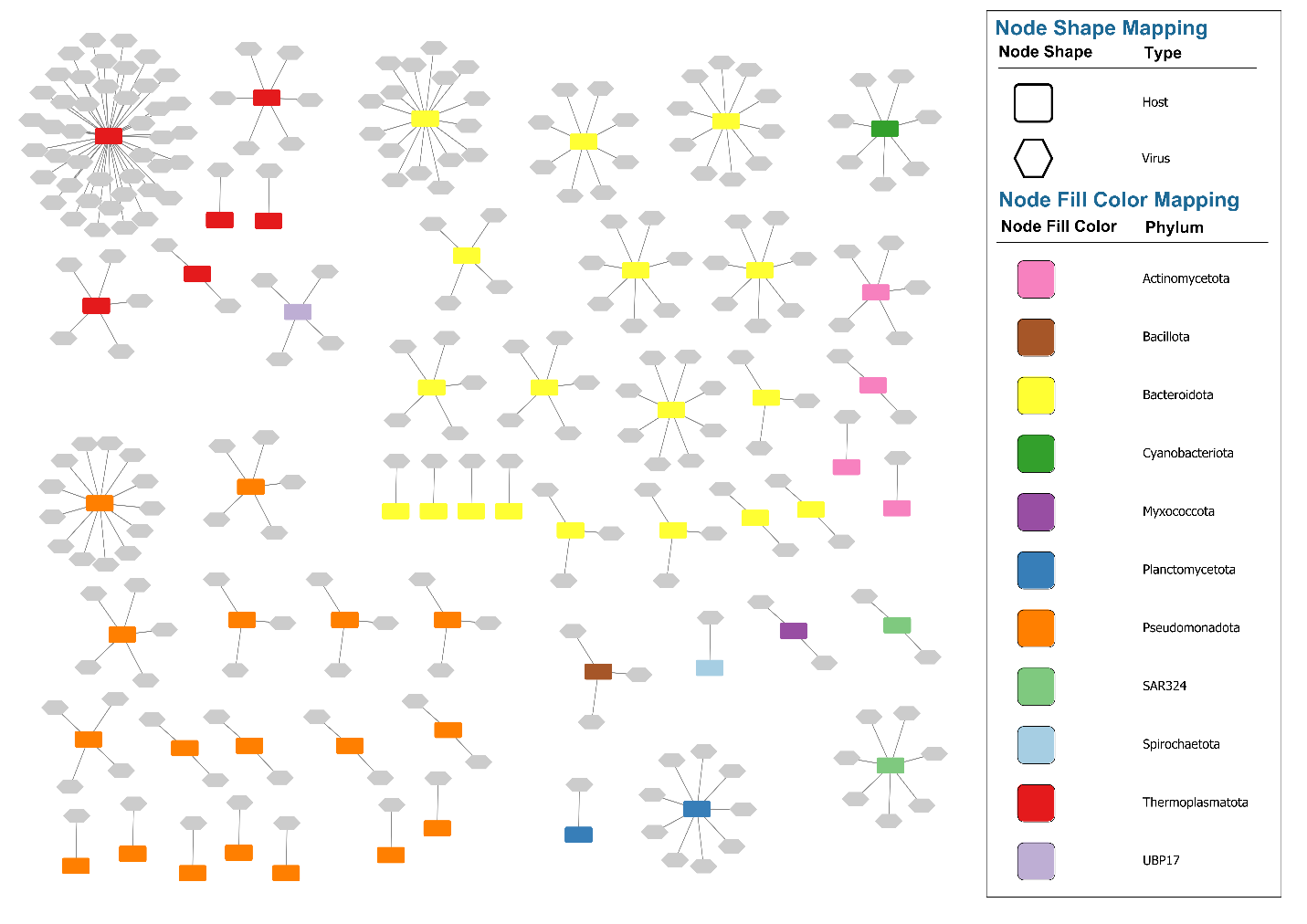


**Figure S10** Network of 241 local virus-host pairs grouped by host phylum, visualized using Cytoscape. Microbial hosts are rectangular while viruses are hexagonal. Each host phylum is assigned a different color.

**Supplementary table legends**

**Table S1** List of keywords for removal during AMG manual curation.

**Table S2** Sampling site conditions. Bottom water samples are indicated with “B” in the sample name. All nutrient concentrations are mg/L.

**Table S3** Sequencing and sequence processing results of microbial and virion fractions.

**Table S4** AMG identification results. “AMG counts” refers to the raw number of AMGs identified; “AMG types” refers to the non-redundant amount of different genes identified.

**Table S5** Conserved AMG comparison: presence of AMGs in Kieft 2020, Heyerhoff 2022, and this study.

**Table S6** List of AMG functions corresponding to the genes in Figure 5.

**Table S7** Type I AMGs unique to the virion fraction. The 8 virion fraction derived AMGs not present in the microbial cellular fraction in Fig. S7 are shown here.

**Table S8** PERMANOVA table to evaluate the relative contribution of host and AMG category in explaining the variation of virus AMG presence. A Bray-Curtis dissimilarity index was used, and 999 permutations were performed.

**Table S9** The summary of the quality of viral populations, generated by CheckV.
