# Supplementary material for: Virus-encoded metabolism may support environmental stress adaptation of microbial hosts in an estuarine hypoxic zone
Source: Front Microbiol. 2026 Mar 24;17:1785655. doi: 10.3389/fmicb.2026.1785655 (PMC13079714; doi:10.3389/fmicb.2026.1785655)
Supplement: Supplementary file 3 [file Data_Sheet_2.docx]

**Data availability**

Raw reads generated in this study have been deposited in the National Center for Biotechnology Information BioProject database with the project ID PRJNA1243077. Microbial metagenome-assembled genomes (MAGs) bins (10.6084/m9.figshare.28740248, https://figshare.com/s/72c9758d779f745bc5e4) and viral populations (10.6084/m9.figshare.28740188, https://figshare.com/s/413254d138c78265cec4) have been deposited on the Figshare website under the project “Microbiome of the Pearl River Estuary summer hypoxic zone”.

Raw sequences for this study have also been deposited in the BMDC National Omics Data Encyclopedia (NODE) under project ID OEP00001662 (https://www.biosino.org/node/project/detail/OEP00001662), with microbial cellular fraction under Experiment ID OEX00031539 and viral fraction under Experiment ID OEX00031538. Assembled sequences are available as analyses including microbial bins (Analysis ID: OEZ00021845) and viral populations (Analysis ID: OEZ00021846).

The quality summary of viral populations is available as Table S9. Amino acid sequences of AMGs are available as supplementary files (0.2_fraction_AMG_aa_sequences.xlsx, Viral_fraction_AMG_aa_sequences.xlsx).

Contents of supplementary data files:

0.2_fraction_AMG_aa_sequences.xlsx

The amino acid sequences of AMGs derived from viruses from the microbial cellular fraction, sorted by metabolic category.

Viral_fraction_AMG_aa_sequences.xlsx

The amino acid sequences of AMGs derived from viruses from the viral fraction, sorted by metabolic category.
